# Topological modelling of urban air pollution and cognition

**DOI:** 10.1038/s44482-025-00009-z

**Published:** 2026-04-08

**Authors:** Holger Engleitner, Marta Suárez Pinilla, Martin Rossor, Parashkev Nachev

**Affiliations:** 1 UCL Queen Square Institute of Neurology, University College London, London, UK; 2 Clinical Neuroscience Laboratory, Centre for Biomedical Technology, Universidad Politécnica de Madrid, Madrid, Spain

**Keywords:** Epidemiology, Epidemiology

## Abstract

The impact of air pollution on cognitive function has attracted repeated scrutiny, and current results point to a potentially detrimental role. The purpose of this study was to examine this association in geographic space. Cross-sectional, complete observational data from UK Biobank were extracted for the four metropolitan regions of Birmingham, Leeds, Liverpool and Manchester in England, UK, including three pollution indicators and two measures of cognitive performance. A set of additional covariates served to adjust for potential confounders. Spatial analyses for each region and combination of pollution indicator and cognition measure were conducted using mass-univariate linear regression in GeoSPM. Conventional non-spatial Bayesian regression models were used for comparison. A significant interaction between air pollution and cognitive performance was identified in 51 areas based on a two-tailed t-test at p < 0.05 FEW (voxel-level family-wise correction). In 29 of those areas, increased pollution and reduced cognition co-occur, and a pattern of central locations and primary roads emerges, suggesting a potentially harmful effect predicated by geography.

## Introduction

There is growing recognition of a potential adverse link between exposure to air pollution and human cognition, supported by a steadily accumulating number of studies^1–3^. Amongst the potential effects of air pollution are structural changes in the neonatal brain^4^, impaired cognitive performance in children as well as adults^5,6^ and the onset of dementia^7^, to name a few recent examples. The connection between air pollution, cognitive decline and dementia in older people has also recently been independently reviewed and acknowledged by an advisory group to the UK government, the Committee on the Medical Effects of Air Pollutants^8^.

Cognitive impairment in general and the specific question of how it can be traced to air pollution refers back to a larger debate about cognitive health in society, in which we have advocated for the notion of a cognitive footprint^9^: As cognitive capital over the human lifespan can be affected by actions and factors (or their absence) in different domains and situations, their collective and cumulative impact on cognitive function should be recognised and accounted for.

Here, we intend to examine the cognitive footprint of air pollution *spatially*: not only is spatial variability a key aspect of environmental exposure that requires appropriate treatment from a methodological point of view, but even more so because of what spatial analysis affords: Insights into patterns, associations and disparities that would be hard to detect and describe otherwise. Beyond its immediate utility for research, where one might be interested in establishing if a spatial pattern is random or represents a substantial deviation from a null hypothesis, spatial analysis is relevant in public health because it can guide policy formation and supply decision makers with actionable evidence. This might be in the form of identifying areas of elevated risk, by examining the spatial structure of environmental hazards or aiding in how resources are allocated based on establishing a spatial distribution of needs. As an added benefit, its visual outputs can help to communicate findings to a wider audience more effectively.

Surprisingly, spatial aspects appear to play a role in only a fraction of studies that examine air pollution and cognition. Based on a series of PubMed queries we conducted on titles and abstracts as of 15 September 2025, out of 2581 articles that matched at least one relevant term for air pollution and cognition, less than 10% also mentioned a term referring to a spatial aspect, for example residential location or terms suggestive of a spatial analysis (a more detailed description is provided in supplementary materials, Tables S.1–S.8).

We identified UK Biobank^10^, internationally the largest multi-modal repository of rich data in public health and medicine, as a suitable resource for this study, since it provides home locations as well as cognitive assessments for its large cohort, and spatially covers several larger cities in the UK. Interestingly, as with the earlier PubMed search, a dearth of spatially oriented analyses seems to persist within UK Biobank, as far as air pollution and cognition are concerned: A PubMed search of “air pollution” and “UK Biobank” in the title or abstract yields 320 articles as of 15 September 2025. 29 studies were concerned with cognition (Table S.9), mainly in terms of dementia, or brain-morphological effects, but none of them had a discernible spatial component—such as spatial covariates, spatial autocorrelation, spatial errors or spatially varying or weighted models—or spatially-structured outputs. Conversely, 11 studies (Table S.10) outside cognition incorporated some spatial aspect as part of their analysis: areal differentiation, heat maps, spatial interpolation or point plots, or geographic maps. As can be seen from Table S.11, UK Biobank is by far the largest cohort among potential cohorts in the UK that hold information on cognition and residential location.

Reaction time is a commonly used measure of cognitive performance, especially in the context of aging and age-related effects, and assumed to be representative of the speed and efficiency of underlying cognitive processes^11,12^. It is known to be susceptible to short term fluctuations: observable changes in this intraindividual variability have garnered interest as a potential indicator of age and cognitive health^13^. For the purposes of this study, with a firmly middle-aged cohort, reaction time seems a suitable measure of cognitive function. In UK Biobank, cognitive performance is assessed by several touchscreen-based tests during the initial assessment centre visit, and we chose mean reaction time to identify a match in a simple visual card game as one of our measures. Because this is the only reaction time-based variable in UK Biobank, we also included completion time for matching memorised pairs in a grid of cards as another speed-dependent variable.

Our aim for this study is to explore the relationship between air pollution and cognition spatially, as this seems to be an aspect that has not been well explored, neither in the context of UK Biobank nor beyond. The objective is to determine whether a significant effect of air pollution on cognition can be ascertained in geographic space while controlling for relevant variables, and to describe its spatial structure in an urban environment, which is where the majority of the world’s population now live.

We conducted our analysis using GeoSPM^14^, a software tool we previously developed for efficient spatial inference on point data. GeoSPM is a genuine spatial method: It relies on the asymptotic topological properties of the geometry of excursion sets of (Gaussian) random fields to strictly control type 1 errors when thresholding the statistic parametric maps of regression coefficients. It trades off the greater expressivity afforded by more powerful non-linear multivariate methods^15^ for greater robustness to noise, conceptual simplicity, modest computational complexity, and flexibility to examine interaction effects, which is the means by which we will determine effect sizes.

To the best of our knowledge, as of 15 September 2025 no previously published study in UK Biobank has explored the relationship between air pollution and cognition using spatial methods or determined the significance of a corresponding spatial effect based on interactions.

## Results

### Main analysis

Computation of the Bayesian multiple regression models with a ridge prior produced the posterior estimates summarised in Tables 1–2, for the effect of pollution on reaction time, and for the effect of pollution on completion time, respectively. In the latter setting, in 11 out of 12 models, pollution shows a significant effect, but the rank within each model is low. In the case of reaction time, the number of models with a significant pollution effect decreases to six out of 12, with equally low model ranks. Unsurprisingly in view of their simplicity, these models do not represent a good fit, leaving most of the variation unexplained. However, they do show an association between cognition and pollution that is quantitively not negligible.

**Table 1:** Posterior estimates of the effect of pollution on reaction time for Bayesian regression models

City	Selected Pollution Measure	Mean	Std	95% CI		t Statistic	Rank in Model	ESS	R^2^ Model
Birmingham	NO_2_	0.033**	0.008	0.017	0.050	4.006	7	99.8	0.122
	NO_x_	0.020**	0.007	0.006	0.035	2.776	10	99.8	0.122
	PM_2.5_	0.016**	0.007	0.001	0.030	2.163	10	99.8	0.122
Leeds	NO_2_	0.011*	0.010	-0.008	0.030	1.120	11	99.8	0.111
	NO_x_	0.003	0.008	-0.013	0.018	0.329	12	99.9	0.111
	PM_2.5_	0.012*	0.010	-0.007	0.030	1.200	11	99.8	0.111
Liverpool	NO_2_	0.012*	0.009	-0.005	0.030	1.416	9	99.8	0.122
	NO_x_	0.011*	0.008	-0.005	0.027	1.358	11	99.8	0.122
	PM_2.5_	0.014*	0.009	-0.003	0.032	1.612	9	99.8	0.122
Manchester	NO_2_	0.039**	0.008	0.024	0.055	4.928	7	99.8	0.124
	NO_x_	0.032**	0.007	0.018	0.046	4.509	8	99.8	0.124
	PM_2.5_	0.039**	0.008	0.023	0.054	4.992	7	99.8	0.124

* indicates the interquartile range of coefficients does not cross zero.

** indicates the 95% interval does not cross zero.

**Table 2:** Posterior estimates of the effect of pollution on completion time for Bayesian regression models

City	Selected Pollution Measure	Mean	Std	95% CI		t Statistic	Rank in Model	ESS	R^2^ Model
Birmingham	NO_2_	0.030**	0.008	0.014	0.046	3.580	7	99.8	0.103
	NO_x_	0.019**	0.007	0.004	0.033	2.538	10	99.8	0.103
	PM_2.5_	0.016**	0.007	0.002	0.031	2.185	10	99.8	0.103
Leeds	NO_2_	0.020**	0.010	0.001	0.039	2.061	9	99.8	0.088
	NO_x_	0.026**	0.008	0.010	0.041	3.147	9	99.8	0.089
	PM_2.5_	0.015*	0.010	-0.004	0.034	1.531	11	99.8	0.088
Liverpool	NO_2_	0.023**	0.009	0.005	0.040	2.534	9	99.8	0.089
	NO_x_	0.024**	0.008	0.007	0.040	2.807	9	99.8	0.089
	PM_2.5_	0.025**	0.009	0.007	0.042	2.760	9	99.8	0.089
Manchester	NO_2_	0.040**	0.008	0.024	0.055	4.940	7	99.8	0.114
	NO_x_	0.030**	0.007	0.016	0.044	4.175	9	99.8	0.114
	PM_2.5_	0.031**	0.008	0.016	0.046	4.016	9	99.8	0.114

* indicates the interquartile range of coefficients does not cross zero.

** indicates the 95% interval does not cross zero.

**Fig. 1:**
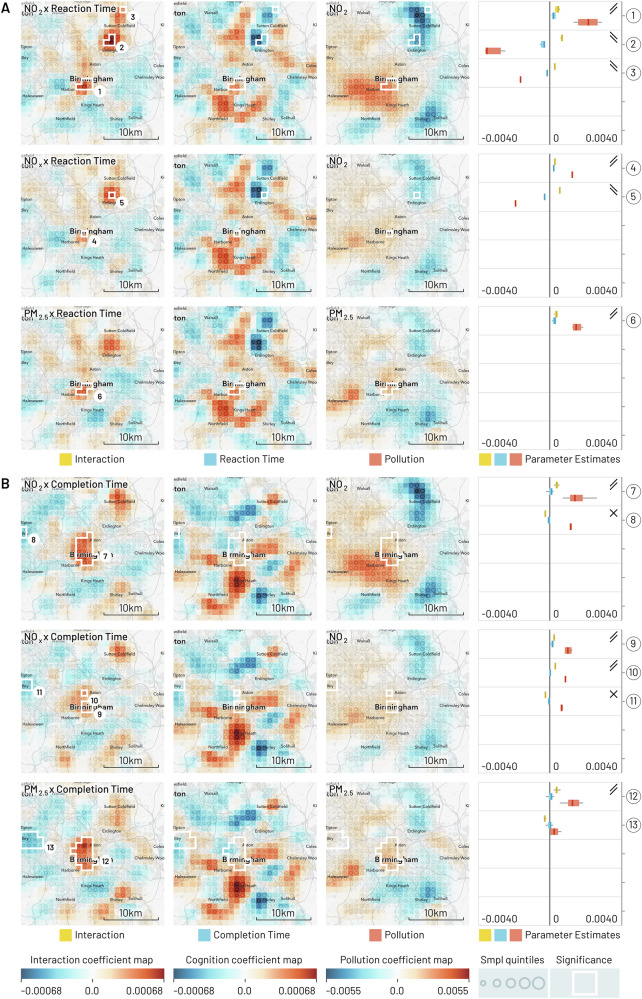
Geographic regression maps showing interaction and individual effects for cognition and air pollution in Birmingham with smoothing applied (95% of kernel density within a 5 km diameter). Each row originates from a separate GeoSPM model comprising a shared set of covariates (not shown here) and a specific combination of cognition and pollution, while each column groups (from left to right) the effects for interaction, cognition and pollution. A colour scale from blue to red has been applied per column. Significant areas of a two-tailed t-test at *p* < 0.05 FEW (voxel-level family-wise correction) with at least 10 participants for the interaction effect are marked by a white border and reproduced in the individual effects. The areas are numbered and referenced in the last column, in which the distribution of coefficients is summarised (min-max, IQR and median) per area, to compare their dominant sign and help in the interpretation of the interaction. Rising double bars indicate an interaction with positive increments for individual effects, falling double bars indicate an interaction with negative increments, crossed bars indicate an interaction with opposite increments, and missing bars indicate inconsistent signs between interaction and individual effects. UK Biobank provide locations at a resolution of 1 km^2^ and each map shows an area of 26 by 26 km. The quintiles of the sample density are indicated by the size of a circle. **A** The cognition variable is reaction time and each row represents a different measure of pollution, from top to bottom: NO_2_, NO_x_ and PM_2.5_. **B** The cognition variable is pair matching completion time with the same measures of pollution as in (**A**).

**Fig. 2:**
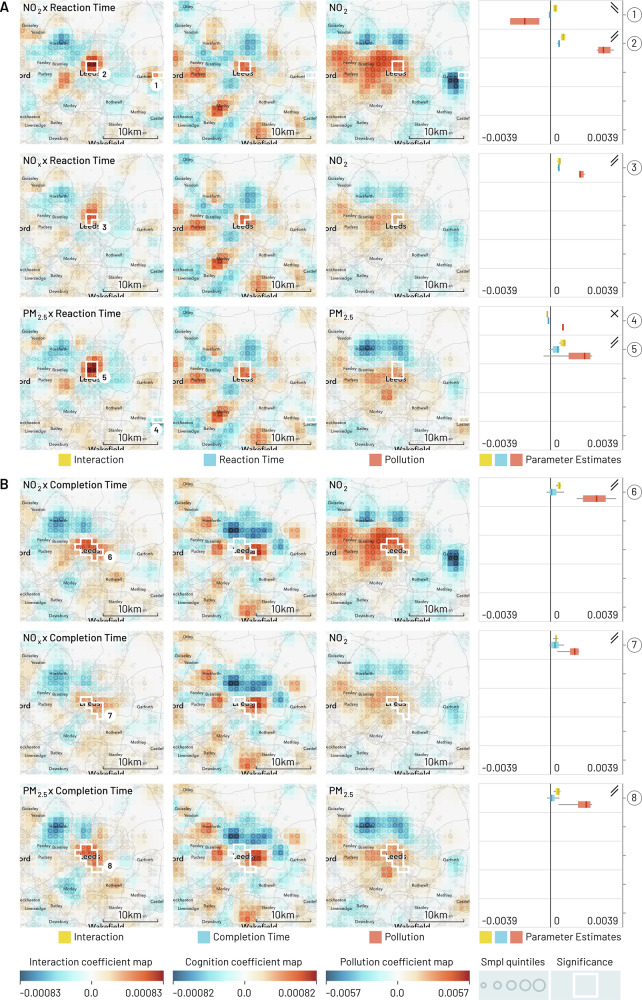
Geographic regression maps showing interaction and individual effects for cognition and air pollution in Leeds with smoothing applied (95% of kernel density within a 5 km diameter). Each row originates from a separate GeoSPM model comprising a shared set of covariates (not shown here) and a specific combination of cognition and pollution, while each column groups (from left to right) the effects for interaction, cognition and pollution. A colour scale from blue to red has been applied per column. Significant areas of a two-tailed t-test at *p* < 0.05 FEW (voxel-level family-wise correction) with at least ten participants for the interaction effect are marked by a white border and reproduced in the individual effects. The areas are numbered and referenced in the last column, in which the distribution of coefficients is summarised (min-max, IQR and median) per area, to compare their dominant sign and help in the interpretation of the interaction. Rising double bars indicate an interaction with positive increments for individual effects, falling double bars indicate an interaction with negative increments, crossed bars indicate an interaction with opposite increments, and missing bars indicate inconsistent signs between interaction and individual effects. UK Biobank provide locations at a resolution of 1 km^2^ and each map shows an area of 26 by 26 km. The quintiles of the sample density are indicated by the size of a circle. **A** The cognition variable is reaction time and each row represents a different measure of pollution, from top to bottom: NO_2_, NO_x_ and PM_2.5_. **B** The cognition variable is pair matching completion time with the same measures of pollution as in (**A**).

**Fig. 3:**
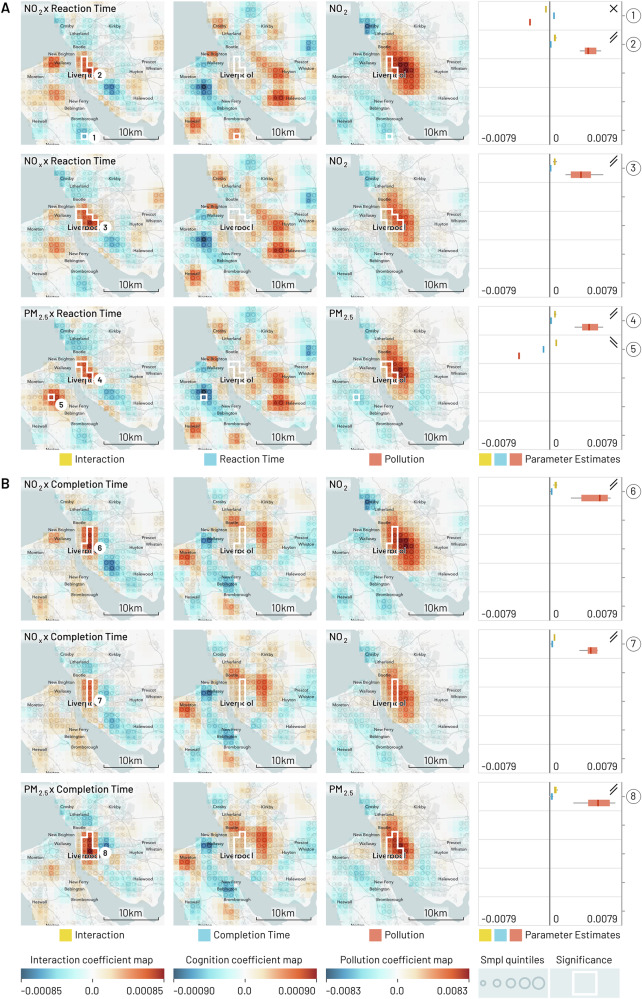
Geographic regression maps showing interaction and individual effects for cognition and air pollution in Liverpool with smoothing applied (95% of kernel density within a 5 km diameter). Each row originates from a separate GeoSPM model comprising a shared set of covariates (not shown here) and a specific combination of cognition and pollution, while each column groups (from left to right) the effects for interaction, cognition and pollution. A colour scale from blue to red has been applied per column. Significant areas of a two-tailed t-test at *p* < 0.05 FEW (voxel-level family-wise correction) with at least ten participants for the interaction effect are marked by a white border and reproduced in the individual effects. The areas are numbered and referenced in the last column, in which the distribution of coefficients is summarised (min-max, IQR and median) per area, to compare their dominant sign and help in the interpretation of the interaction. Rising double bars indicate an interaction with positive increments for individual effects, falling double bars indicate an interaction with negative increments, crossed bars indicate an interaction with opposite increments, and missing bars indicate inconsistent signs between interaction and individual effects. UK Biobank provide locations at a resolution of 1 km^2^ and each map shows an area of 26 by 26 km. The quintiles of the sample density are indicated by the size of a circle. **A** The cognition variable is reaction time and each row represents a different measure of pollution, from top to bottom: NO_2_, NO_x_ and PM_2.5_. **B** The cognition variable is pair matching completion time with the same measures of pollution as in (**A**).

**Fig. 4:**
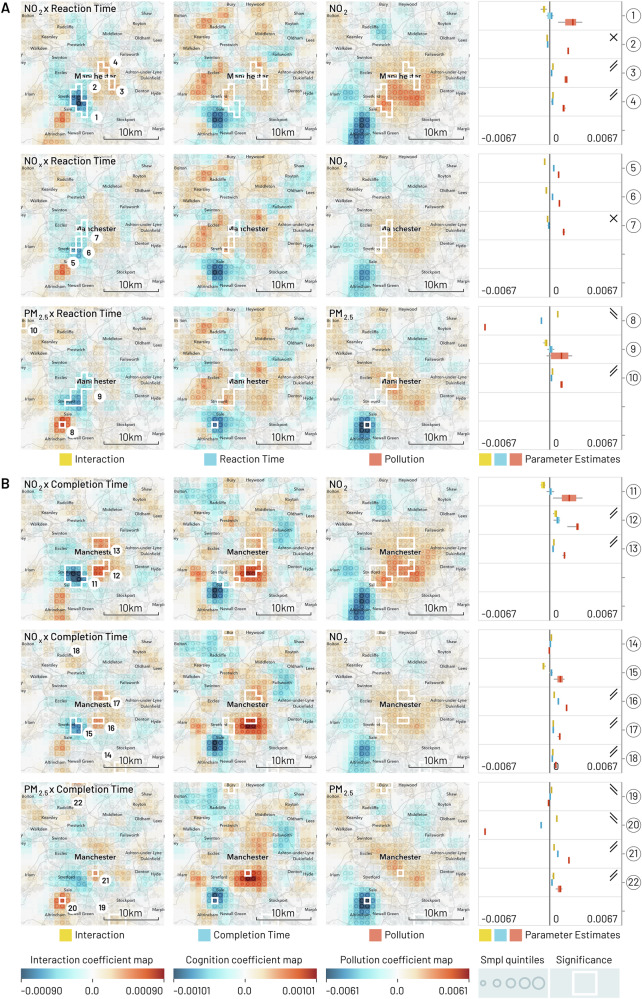
Geographic regression maps showing interaction and individual effects for cognition and air pollution in Manchester with smoothing applied (95% of kernel density within a 5 km diameter). Each row originates from a separate GeoSPM model comprising a shared set of covariates (not shown here) and a specific combination of cognition and pollution, while each column groups (from left to right) the effects for interaction, cognition and pollution. A colour scale from blue to red has been applied per column. Significant areas of a two-tailed t-test at *p* < 0.05 FEW (voxel-level family-wise correction) with at least ten participants for the interaction effect are marked by a white border and reproduced in the individual effects. The areas are numbered and referenced in the last column, in which the distribution of coefficients is summarised (min-max, IQR and median) per area, to compare their dominant sign and help in the interpretation of the interaction. Rising double bars indicate an interaction with positive increments for individual effects, falling double bars indicate an interaction with negative increments, crossed bars indicate an interaction with opposite increments, and missing bars indicate inconsistent signs between interaction and individual effects. UK Biobank provide locations at a resolution of 1 km^2^ and each map shows an area of 26 by 26 km. The quintiles of the sample density are indicated by the size of a circle. **A** The cognition variable is reaction time and each row represents a different measure of pollution, from top to bottom: NO_2_, NO_x_ and PM_2.5_. **B** The cognition variable is pair matching completion time with the same measures of pollution as in (A).

For GeoSPM, there were also 24 models in total, one model for each combination of the cognition and pollution variables across the four cities. The corresponding regression coefficient maps are reproduced in Figs. 1–4 for each city. Significant regions were determined by a two-tailed t-test at *p* < 0.05 FWE (voxel-level family-wise correction) and omitted when they included less than 10 participants in the underlying cohort (of which there were only a few). The square areas span 26 by 26 km at the original 1 km^2^ resolution of the data and reflect the uneven distribution of samples in UK Biobank.

These maps present a spatially de-confounded view of the interaction between cognitive performance and air pollution as far as the other covariates are concerned. We obtain regions of significant interactions for every single combination and city. Out of 51 regions in total, the interaction is positive and the signs of the individual effects afford a consistent interpretation in 37: In 29 of these areas (Birmingham: 1, 4, 6, 7, 9, 10, 12; Leeds: 2, 3, 5, 6, 7, 8; Liverpool: 2, 3, 4, 6, 7, 8; Manchester: 3, 4, 10, 12, 13, 16, 17, 18, 21, 22), we find above average pollution and reduced cognition, while in 8 areas (Birmingham: 2, 3, 5; Leeds: 1, Liverpool: 5, Manchester: 8, 19, 20), below average pollution co-occurs with increased cognition. Another 6 of the 51 regions show negative interactions, where the direction of individual effects is in opposition and the interpretation of the modifying effect of pollution is counter-intuitive, so that above average pollution is spatially associated with increased cognition and vice-versa (Birmingham: 8, 11; Leeds: 4; Liverpool: 1; Manchester: 2, 7). The remaining group of eight regions does not show consistent signs across the different effects, so their interpretation in aggregate is inconclusive: 7 of those are located in Manchester (1, 5, 6, 9, 11, 14, 15) and one in Birmingham (13).

The strongest pattern emerges for Leeds, Liverpool—and to a slightly lesser degree—Birmingham, where a positive interaction between reduced cognition and increased pollution persists in mainly central areas and for all three pollutants. The pattern applies to both reaction and completion time: in Leeds, the extent of the effect is weaker for reaction time, while in Birmingham the distinction is even more pronounced, in extent as well as localisation. For the latter city, we can also distinguish more peripheral areas showing an inversion of the pattern: increased cognition appears alongside reduced pollution.

Manchester reveals a more heterogeneous structure: an increase in variation between NO_2_, NO_x_ and PM_2.5_, a tendency towards the periphery, two regions—both for reaction time—where the interaction is negative and the interpretation counter-intuitive, and seven regions without consistent signs between interaction and individual effects. Notable among these areas without a conclusive interpretation is a set of regions with a large share of samples (reaction time: 609 for NO_2_, area 1; 599 for PM_2.5_, area 9; completion time: 889 for NO_2_, area 11; 385 for NO_x_, area 15) that roughly coincide with the suburb of Chorlton-cum-Hardy in the southwest. There, the interaction is negative, both pollution measure effects are positive, but the area straddles cognition effects that are either fully or partially positive. For area 9, this is clearly an artefact of two smaller significant interaction areas in direct adjacency: when looking at the individual effects for the northern and southern half in isolation, the signs are consistent.

Nonetheless, the pattern of reduced cognition and increased pollution observed in the centre of the other three cities survives somewhat diminished in Manchester for completion time and NO_2_ and NO_x_, and again for NO_2_ and reaction time. In addition, the inverse relationship of increased cognition and less pollution manifests approximately in Brooklands, southwest of Manchester. The corresponding area contains 150 samples for PM_2.5_ (area 8) and reaction time and PM_2.5_ and completion time (area 20).

Tables S.15 and S.16 summarise parts (A) and (B), respectively, of Figs. 1–4, providing the number of the region in the figures, number of participants (N) and 1 km^2^ cells (C), as well as place labels (where possible) from Open Street Map data.

### Sensitivity Analyses

The robustness of these results in terms of our choice of parameters—kernel size, region size and region location—are demonstrated by the sensitivity analyses reported in supplementary materials in Figs. S3–S23.

In general, as kernel size varies, significant areas remain located around the same positions, while their spatial extent and contiguity changes, as expected: The choice of kernel size involves a trade-off between sensitivity and spatial resolution, but does not impact the validity of the results. The full range of kernel sizes is reported only for Liverpool (Figs. S3–S9), whereas two kernel variations are reported for Birmingham (Figs. S10 and S11), Leeds (Figs. S12 and S13) and Manchester (Figs. S14 and S15) in the interest of brevity.

Similarly, the results of the primary analysis reported here are stable with respect to an enlarged size of the analysis region, as can be seen in Figs. S16, S18, S20 and S22. Minor variations do occur, which is not surprising, giving that the cohorts for three out of four regions increase considerably (Leeds 44%, Liverpool 32%, Manchester 38%).

A greater impact on the stability of results can be observed when the area of analysis is shifted, which is in line with our expectation, as the composition of each cohort underlying the shifted area changes considerably: Isolated single-voxel areas might be lost, and the boundaries of larger clusters might vary. Nevertheless, positions remain stable, and areas maintain similarity to their unshifted analogues (cf. Figs. S17, S19, S21 and S23).

We observe that in the reduced cohort case, which only includes participants that remained at the same residential address at the 1 km by 1 km grid cell level, results are largely comparable to the main results reported here. The similarity is greater for Leeds and Manchester, and less pronounced for Liverpool and Birmingham, especially with reaction time. The reduction in cohort sizes is substantial, amounting to 25% per city on average.

## Discussion

We examined the spatial association between cognitive performance (reaction time and completion time) and air pollution (NO_2_, NO_x_ and PM_2.5_) in Birmingham, Leeds, Liverpool and Manchester by estimating the corresponding interaction and individual effects in a spatially de-confounding GeoSPM regression model, using non-spatial Bayesian regression models for comparison. Our study found positive interactions between increased pollution and reduced cognition in central areas of all four cities and for all three pollutants. For a small number of mostly peripheral instances this relationship is expressed in its inverted form, indicating a benefit of cleaner air for increased cognition. Some areas where the interaction cannot be easily interpreted were also identified as statistically significant: a negative interaction is counter-intuitive, because it suggests that pollution has a beneficial effect on cognition or that the lack of it is cognitively detrimental, contrary to the biological evidence^16,17^. This suggests hidden confounders not captured in the model. An inconclusive interaction on the other hand has more local variability in terms of its individual effects and their signs, so that the region, while still statistically significant, has no unique interpretation *in aggregate*: The interaction can still be interpreted for each grid cell separately. Such an interaction can sometimes indicate a structure of subregions with consistent interpretations that happen to be in proximity. In both cases, further investigation is merited, but outside the scope of this analysis. The detection of positive interactions between increased pollution and reduced cognition is consistent with previous findings reported in the literature (see the reviews by Thompson et al.^1^ and Delgado-Saborit et al.^3^).

A recent study^18^ of the French CONSTANCES cohort used a non-spatial regression analysis between cognitive performance and air pollutants of a sub-sample of 61462 participants 45 years and older: Their results showed significant associations of worse cognitive function and increased pollution, mostly for black carbon and NO_2_, whereas exposure to PM_2.5_ was mainly associated with decreased performance in a semantic fluency test in suburban areas. The comparison to this work seems particularly relevant. CONSTANCES and UK Biobank share a similar approach in their design. They are based on a random sample derived from health care records, with a stratified sampling scheme to be representative of the local population, and enrolment through several regional assessment centres. In both studies, cohorts are middle-aged and large, exposure was assessed for a 2010 baseline based on similar LUR modelling and used a participant’s residential address at the time of enrolment, with an overlapping set of air pollutants (NO_2_ and PM_2.5_). The varying results of stratified analyses based on four categories of residential area in the CONSTANCES study hint at the importance of the spatial context in this type of analysis. Key differences to this study concern the imputation vs exclusion of missing data and the age cut-off of people over 65, based on the assumption that with age the likelihood of pathological decrease in cognitive function increases.

Another recent study by Wood et al.^19^ examined air pollution and cognition in national and London-only cohorts (*n* = 8883 and *n* = 768) derived from the English Longitudinal Study of Ageing (ELSA) using linear mixed-effects models. They found significant decreases in test scores of composite memory and executive function when increasing long-term exposure for NO_2_, PM_2.5_, and PM_10_ based on repeated cognitive measurements. Exposure assessment employed annual average concentrations from the 2012 Community Multiscale Air Quality Urban dispersion model and the assignment to participants’ postcodes was based on deciles due to privacy concerns. Address changes were accounted for by assigning pollution estimates based on the year of the (follow-up) interview and the postcode available for that year.

A Bayesian spatial survival analysis of a smaller cohort (*n* = 1572) is reported by Sullivan et al.^20^ for the Monongahela valley of south-western Pennsylvania in the United States, a region with an industrial history of steel production and high air pollution. Sullivan et al.^20^ investigated the relationship between PM_2.5_ exposure in later life and the incidence of mild cognitive impairment as well as dementia among a community-based sample of adult, mainly life-long, residents of this region. They found significant higher adjusted risk of incident mild cognitive impairment and dementia for increases in PM_2.5_ assessed at the census-tract level. The interest here lies in the fact that this relationship was detected by a spatial analysis based on a much smaller sample, and the overlap with our results for PM_2.5_.

For a Chinese cohort (*n* = 15163), Gao et al.^21^ analyse the effect of PM_2.5_, NO_2_ and O_3_ on cognitive function, including a spatial modelling approach using a spatial error model, which treats spatial correlations as a nuisance factor (they determined a low level presence of spatial correlation in the data). They apply a more basic exposure assessment solely based on kriging of 1389 air monitoring sites across China for annual pollution concentrations. Their results show PM_2.5_ as the dominant air pollutant affecting cognition over 1-, 2-, 3- and 4-year exposure periods, while NO_2_ only produced a significant effect at the 4-year period, which was more significant than the corresponding 4-year PM_2.5_ effect.

Interestingly, a non-spatial study (Cullen et al.^22^) based on the same UK Biobank dataset underlying this work, only found small negative relationships between pollutant exposure (PM_2.5_, PM_10_, PM_2.5–10_, NO_2_ and NO_x_) and cognitive test performance in unadjusted regression models. These associations shrank further and became inconsistent in terms of direction, when adjusted for confounders. Out of 25 regression models, only 5 had a p-value less than 0.05 (false discovery rate), and 2 of those showed a counter-intuitive direction of the pollution effect on a reasoning score. The other 3 significant results showed significant effects for PM_2.5_ and NO_x_ on reaction time and for NO_2_ on pairs matching completion time, which were found to be consistently significant in this work, as described above. Cullen et al.^22^ do not consider changes in address for their main analysis but found slightly stronger associations in their sensitivity analysis using alternative exposure measurements that included the history of residential addresses for each participant. The number of observations per analysis varied but was over 70000 for reaction time and pairs matching completion time for PM_2.5_, NO_2_ and NO_x_. These results contrast with our findings here and could be an indication that the spatial nature of exposure (and potentially other covariates) cannot be ignored.

Local geography offers additional context for the interpretation of individual regions for which a co-occurrence of reduced cognition and increased pollution was found, even if the coarse spatial resolution provided by UK Biobank renders matching effects to local features difficult. However, we can constrain ourselves to an examination of the primary road structure, as a major source of the pollutants discussed here is road traffic.

**Fig. 5:**
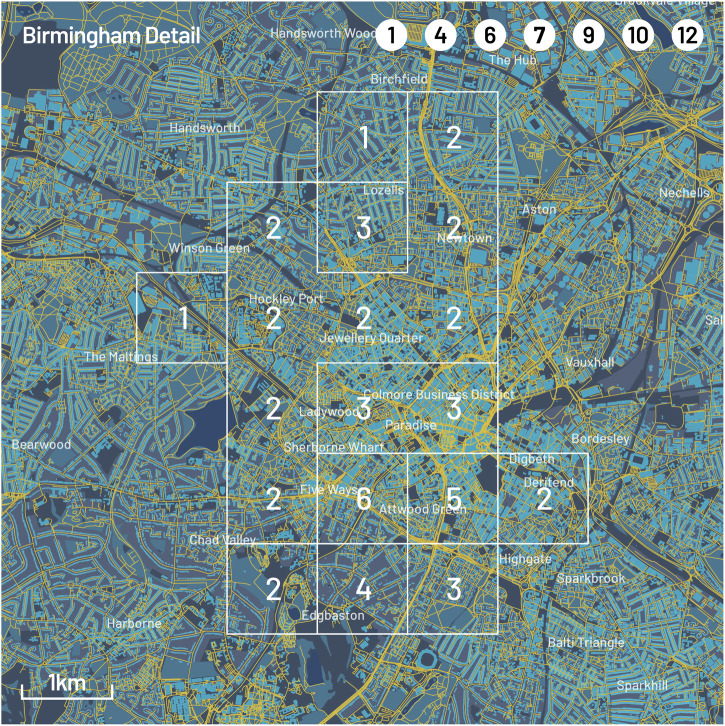
Detail of geographic context for Birmingham for a subset of the significant areas shown in Fig. 1. Their identifying numbers are listed in the top right and their outlines delineated in white. All regions exhibit a positive interaction and individual effects that indicate the co-occurrence of reduced cognition and increased air pollution. Each grid cell indicates the count of significant areas it is part of. Grid cells with higher counts contain primary roads and are located in central areas. Geographic data provided by OpenStreetMap.

In Birmingham, we see that significant grid cells tend to concentrate in the city centre and in proximity to primary roads (Fig. 5). Most of the A4540 Middleway ring road appears in regions 7, 9 and 12 for completion time, as well as the inner A38 ring road and its Queensway section that flank the historic centre. The southern part of the A4540 near Attwood Green also appears in regions 1, 4 and 6. These roads have been previously identified by the UK government as exceeding emission limits in its 2017 air quality plan for nitrogen dioxide in UK (p. 13)^23^, with modelled concentrations ranging between 41–60 µgm/m^3^ for most sections, and a part of Queensway in exceedance of 60 µgm/m^3^. Measured pollution levels matched these exceedances, as published in a status report by Birmingham city council in 2019^24^. The considerable majority of stations along those main roads reported measurements in the range between 40–79 µgm/m^3^. This is acknowledged in the report’s conclusion, stating that “The City continues to have air quality breaches against the annual mean objective for NO_2_ with known exceedance areas being within the city centre. The primary source of air quality issues within Birmingham is road transport.” A similar statement can be found in the city’s Air Quality Action Plan 2021–2026^25^. Birmingham implemented a clean air zone inside the Middleway ring road, excluding the road itself in June 2021^26^. A recent assessment of the change concluded significant but modest reductions in NO_2_ and NO_x_ concentrations, but none for PM_2.5_^27^. In view of the exceedances reported by Birmingham council, it is important to remember that the World Health Organisation updated their air quality guidelines in 2021^28^ with stricter limits than the air quality objectives currently mandated in the UK: The new recommendations are 10 µg/m^3^ for NO_2_ (currently 40 µg/m^3^) and 5 µg/m^3^ for PM_2.5_ (currently 20 µg/m^3^, 12 µg/m^3^ from 2028).

Additional discussion of the geographic context for the other cities is provided in Supplementary Materials (Figs. S28–S30) and shows comparable correspondences.

The results of this study contribute to the growing signs that indicate a negative effect of air pollution on cognition: at the policy level, these findings underline the continued, pressing need to reduce emissions of air pollutants because of their cognitive footprint. A diminishment of cognitive capital because of factors that can be controlled through policy is not only devastating from an individual perspective but should be avoided for its negative repercussions from a societal and economic perspective: Increased cognitive impairment, especially in old age, puts additional strain on health care systems and families. Separately, in the context of a global transition to knowledge-based economies, societies through their policies make a choice about how much they value the collective cognitive capital of their populations. Independently, the contrast between central and peripheral areas and the change in interpretation of the effect hints at an inequality of the burden of air pollution in terms of its geographical distribution that must be of concern to policy makers. At a technical level, the presence of spatial structure in the relationship between air pollution and cognition implies the need to consider spatially informed approaches to analysis, as non-spatial approaches might underestimate or even fail to detect associations mediated in space. In addition, a finer grained mesh of measurement sites – not only along major traffic routes but also in residential areas and other areas of interest – could give future studies an immense boost in spatial precision.

The strength of this study lies in applying a spatial methodology to a large sample size, which allows to analyse multiple cities in a shared geographic context and enables observations of a common spatial pattern—including centrality and correlations with the primary road network—addressing an underrepresented aspect in the existing literature.

Some limitations must be considered. The group most affected by air pollution, babies and small children, is regrettably not represented in UK Biobank at all, so the conclusions here are not transferrable to that age group. Evidence of selection bias in UK Biobank has been reported in the literature^29^: participants appear to be healthier and live in less socio-economically deprived areas than non-participants. This suggests that our results are more likely to underestimate potentially harmful interactions. Nevertheless, we expect our results to be generalisable to other comparable datasets given the size of the sample, the stratification of UK Biobank’s recruitment, and the direction of selection bias. Our analysis could also be improved with a more detailed approach to accounting for exposure instead of relying on a fixed baseline of pollution at a single address. Lastly, although sex is a variable in this analysis, no conclusions can be drawn about pollution-cognition interaction differences between sexes. This would require a comparison between the interaction-related effect maps produced by separate analyses for each sex and could be an important component of future studies, as other work in this area seems to suggest^30–32^.

We regard this study as a first step towards determining the cognitive footprint of air pollution, and we hope it can provide additional impetus to research its effect on cognition in more detail, using finer grained and more complex means of analysis.

## Methods

### Study design and participants

The cohort used for this study is from UK Biobank, which holds a wide range of health-related information from over half a million participants recruited across the United Kingdom between 2006 and 2010. Potential participants were invited based on their age (40–69 years) and reasonable travel distance from one of the 22 assessment centres through the National Health Service (NHS)^33^. The baseline visit at an assessment centre required participants to give their written consent, complete a touch-screen questionnaire, take part in a brief interview, have their physical measurements taken and provide samples of their blood, urine and saliva. UK Biobank holds the sex of participants as recorded by the NHS but allows corrections upon request.

Crucially, UK Biobank provides residential location information for each participant, derived from the address the recruitment invitation was sent to, and geocoded to a reference grid with a cell size of 1 km^2^. A higher resolution level of 100 m^2^ is available, but access is only granted on a case-by-case basis and when deemed essential for the purposes of a study. For this reason, the present analysis was performed at the lower resolution level.

Participants are primarily clustered around major cities in Great Britain, and we choose four cities based on their population size, comparable geographic extent and large UK Biobank participation rate: Birmingham, Leeds, Liverpool and Manchester. All four have familiar histories of heavy industry beginning with the Industrial Revolution, followed by industrial decline and modern regeneration based on service and information-oriented economic activity. To facilitate inter-city comparisons, we adopted a common spatial scale and settled on a square analysis region spanning 26 km by 26 km, centred at the geographic centre of each city, which allowed us to capture as many participants as possible while maintaining a coherent geographic context for each place. A map of the four study regions within the UK is shown in Figure S1.

Out of a total number of 502543 in the full UK Biobank cohort we extracted 17,829 participants (*f* = 9945, m = 8275) in Birmingham, 19161 (*f* = 10760, m = 8401) in Leeds, 15830 (*f* = 8837, m = 6993) in Liverpool and 19601 (*f* = 10,474, m = 9127) in Manchester without missing values at baseline. Participants with extreme (outside the 1 or 99th percentile) or missing values were removed from their respective region. A diagrammatic overview of the study design is shown in Fig. S2.

The full set of variables included in the analysis and their derivation is shown in Table S.12 and comprises two measures of cognitive performance (reaction time and pair matching completion time), three different indicators for air pollution (NO_2_, NO_x_ and PM_2.5_, based on estimates for 2010) as well as demographic and non-demographic factors of potential relevance. The characteristics of participants in the four city sub-cohorts are shown in Table S.13, environmental variables are summarised in Table S.14. UK Biobank stratified key demographic parameters (age, sex and postcode as a measure of social deprivation) at the invitation stage, over-sampling certain groups as deemed required^33^. No sampling was applied to the data other than through geographic location and excluding missingness. In all four city-based sub-cohorts, there are slightly more women than men. Further details on how variables were collected by UK Biobank are available at the online showcase (https://biobank.ndph.ox.ac.uk/showcase/). A detailed description of the UK Biobank enrolment process is described in Sudlow et al.^10^.

### Exposure assessment

We chose annual average concentration estimates of NO_2_, NO_x_ and PM_2.5_ as pollution indicators because of their suspected harmful influence on cognitive health^34^. Particulate matter smaller than 2.5 µm is considered particularly harmful because it can cross the alveolar-capillary barrier^35^.

UK Biobank linked each participant’s home address to estimates from land use regression (LUR) models developed by the ESCAPE (European Study of Cohorts for Air Pollution Effects, http://www.escapeproject.eu/) project based on monitoring in the year 2010 (https://biobank.ndph.ox.ac.uk/ukb/ukb/docs/EnviroExposEst.pdf)^36,37^. These models predict pollutants using traffic intensity, topography and land use among other factors.

A known limitation of ESCAPE estimates exists for particulate matter: These estimates are considered reliable only up to a distance of 400 km from the original monitoring area in Greater London, so measures for 33,935 addresses beyond this cut-off are coded as missing in UK Biobank. However, all four cities are within the given distance and therefore not affected.

Accurate exposure assessments are difficult to achieve, so we follow the common practice of using the environmental pollution at a participant’s residential address at baseline as an approximation, which is naturally limited. Other contributing individual factors are not considered. We do not take changes of residential address into account for the main analysis but consider its impact in a sensitivity analysis. Our motivation in doing so stems from our focus on the spatial aspect of the analysis and maximising the available sample.

### Statistical analysis

The variables in Table S.12 were selected from a larger set based on minimising co-linearities while retaining those that showed a degree of correlation with our cognitive variables of interest—reaction time and completion time—by inspecting a covariance matrix. We excluded fluid intelligence as a variable of interest because of its significantly lower coverage in UK Biobank compared to the other two measures (*N* = 221,104 vs. *N* = 496,765 for reaction time and *N* = 498,838 for completion time). All non-binary variables, discrete or continuous, were standardised (µ = 0, std = 1) prior to their evaluation in any of the analyses, except for the cognition time variables, which we transformed via ordered quantile normalisation^38^.

As a benchmark for the spatially confounded case, we assessed the association between cognition and the other covariates, including pollution, by performing Bayesian multiple regression with a ridge prior with reaction time or completion time as the response for each of the cities. The predictors in these models were age, sex, ethnic minority, formal qualifications, subjective health score, basal metabolic rate, alcohol consumption level, anxious feelings, walking pace, frequency of social visits, greenspace percentage and one of the three pollution measures: NO_2_, NO_x_ or PM_2.5_. Our tool for evaluating these models was the MATLAB version of BayesReg (version 1.9.1), which evaluates the required Markov chains using Gibb’s sampling. The posterior computations for each chain were based on 20,000 samples, after discarding an initial 100,000 samples for burn-in and thinning a further 100,000 samples by a factor of 5.

Many observable features in epidemiology, environmental medicine, healthcare policy, and public health have a natural expression in a spatial frame of reference and could be described by a joint spatial distribution. We all live, work, exist *somewhere*, after all, and our biological and behavioural characteristics interact with and are shaped by the physical environment that surrounds us. The analysis of spatially varying or spatially confounded associations between these features, observed at discrete points, necessitates suitable methods that take account of spatial correlations, are suitable for surfacing spatial structure and are robust when faced with noise and sparseness. The method we are employing here, GeoSPM, offers interpretability, flexibility, robustness and does not require intricate parametric specification, instead operating on a fixed assumption of Gaussian random fields. Its relative simplicity and accessibility contrasts with the greater complexity of multivariate spatial methods and thus is particularly suitable for exploring datasets in a spatial manner.

GeoSPM (https://github.com/high-dimensional/geospm) is our previously published extension of SPM12 to the geo-spatial domain. SPM12^39^ (https://www.fil.ion.ucl.ac.uk/spm/) is the dominant regression analysis framework for spatial inference in brain imaging that has found wide-spread use over a period of more than 30 years. GeoSPM makes point-based data amenable to the mass-univariate paradigm of SPM12. It estimates the regression of a local response on a global matrix of predictors, which yields maps of localised parameter estimates that give an indication of the marginalised distribution of the underlying covariate. A key feature of this approach is the topologically derived correction for multiple comparisons when testing regression coefficients or their contrasts. It allows to identify the spatial extent of significant parameters in a controlled manner. Additional information about the wider context of statistical analysis in neuroimaging can be found in the provided references^40–42^.

In GeoSPM, sample concentration takes up the role of the response. The sample concentration for each observation in GeoSPM is derived by placing a Gaussian kernel of pre-determined size at its location. For the present study, we concentrated 95% of the kernel density within a radial diameter of 5 km. At each location, this arrangement of the response can be interpreted as an indicator for the closeness of each individual observation to that point in space: the further away an observation occurred from a particular location, the lower the response.

As in a conventional regression model, we use interaction terms to examine any modifying effects between the covariates we are interested in – air pollution and cognition. A significant interaction in GeoSPM suggests a form of dependence or association of covariates within the spatial setting. The sign of the interaction indicates the direction of the association: for example, a positive interaction indicates either more pollution and reduced cognition or less pollution and increased cognition in a place. To distinguish between the two, we also examine—within the same region—the signs of the corresponding individual effects in aggregate. In cases where the signs of the individual effects are not consistent with the sign of the interaction, we refrain from such an interpretation. The inconsistency can arise because there is no constraint on the sign of effects, but also because the signs within individual effects can vary, as the region we examine is taken from the interaction. In practice, we find that signs remain largely homogenous and consistent. We determine the sign of a region based on its median.

Our model structure for GeoSPM follows from the preceding observation about interactions. With the stated objective of examining cognitive performance and air pollution spatially—while controlling for plausible confounders available to us in UK Biobank—we extend the shared set of covariates used in the Bayesian regression models with two additional covariates for cognition and pollution, as well as interaction terms for the cognition covariate and each of the other covariates.

We thus obtained six models per city that only differ in the respective cognition and pollution variables (either reaction time or completion time, and one of NO_2_, NO_x_ or PM_2.5_) and the corresponding cognition interactions but are otherwise identical. GeoSPM produced estimates for the regression coefficient maps as well as corresponding maps of two-tailed t-tests for these coefficients. GeoSPM determines the significance of these maps using its voxel-level family-wise correction for multiple comparison based on the topology of a Gaussian random process, as outlined earlier, and our chosen level of significance. For each significant region, place names were retrieved from a list of labels extracted from Open Street Map data and a summary describing the size and number of participants in it was automatically generated.

### Sensitivity analysis

We conducted a series of sensitivity analyses to gauge the effect of the kernel size, region size and region location on the results of the main analysis for all four cities. We varied kernel size from 1.25 km to 10 km in 1.25 km steps, to gain insight into the robustness of significant areas reported for the chosen kernel size of 5 km. In addition, for each city, we examined two alternative study regions: an expanded region spanning 36 km by 36 km that contains the primary region and adds a buffer of 5 km in each cardinal direction around it and a translated region, of the same size as the primary region. The shift consisted of a translation of 8 km in the north and east directions—roughly a third of the corresponding edges of the primary region—and represents a trade-off between maintaining enough overlap with the primary region and injecting a meaningful change in position.

Another aspect that requires consideration is the effect of changes in residency. For the main analysis, we included all participants regardless of their residential history with their baseline address. To evaluate how changes in residency affect the main results, we conducted a separate analysis for each city in which participants with changes in their residential address were excluded.

## Supplementary information

Supplementary Information

## Data Availability

The data analysed in this study is available on application to UK Biobank.

## Code availability

The open-source software implementation of GeoSPM used in this study is available on GitHub: https://github.com/high-dimensional/geospm (10.5281/zenodo.7258970).
