# Supplementary material for: Topological modelling of urban air pollution and cognition
Source: NPJ Digit Public Health. 2026 Apr 8;1(1):7. doi: 10.1038/s44482-025-00009-z (PMC13061615; doi:10.1038/s44482-025-00009-z)
Supplement: Supplementary file 1 — Supplementary Information [file 44482_2025_9_MOESM1_ESM.pdf]

Supplementary Material

Table of Contents

2 Table S.1 Number of results for topical PubMed queries and their conjunctions as of 15 September 2025

2 Table S.2 Spatial query, part 1: simple search terms.
3 Table S.3 Spatial query, part 2: combined search terms.
3 Table S.4 Cognition query: search terms.
4 Table S.5 Location query, part 1: simple search terms.
4 Table S.6 Location query, part 2: combined search terms.
5 Table S.7 Pollution query, part 1: simple search terms.
5 Table S.8 Pollution query, part 2: exposure terms query.

6 Table S.9 Previous studies of UK Biobank data involving air pollution and cognition
7 Table S.10 Previous studies of UK Biobank data involving air pollution and incorporating a spatial aspect

7 Table S.11 Cohorts identified for the United Kingdom that contain cognitive and residential information.

8 Table S.12 Variables derived from UK Biobank.
9 Table S.13 Descriptive statistics of participants by city.
10 Table S.14 Descriptive statistics of environmental variables by city.

10 Table S.15 Summary of all significant areas for the interaction between reaction time and each pollution variable and city as shown in Figures 1–4 (A).

11 Table S.16 Summary of all significant areas for the interaction between completion time and each pollution variable and city as shown in Figures 1–4 (B)

12 Figure S.1 Location of study regions within the United Kingdom

13 Figure S.2 Cohort selection and study design.

14–20 Sensitivity Analyses: Kernel Sizes Liverpool – Figures S.3 – S.9
21–22 Sensitivity Analyses: Kernel Sizes Birmingham – Figures S.10 and S.11
23–24 Sensitivity Analyses: Kernel Sizes Leeds – Figures S.12 and S.13
25–26 Sensitivity Analyses: Kernel Sizes Manchester – Figures S.14 and S.15

27–28 Sensitivity Analyses: Region Size and Position Birmingham – Figures S.16 and S.17
29–30 Sensitivity Analyses: Region Size and Position Leeds – Figures S.18 and S.19
31–32 Sensitivity Analyses: Region Size and Position Liverpool – Figures S.20 and S.21
33–34 Sensitivity Analyses: Region Size and Position Manchester – Figures S.22 and S.23

35 Reduced Cohort: Birmingham – Figure S.24
36 Reduced Cohort: Leeds – Figure S.25
37 Reduced Cohort: Liverpool – Figure S.26
38 Reduced Cohort: Manchester – Figure S.27

39 Geographic Context for Leeds
40 Geographic Context for Liverpool
41 Geographic Context for Manchester
42 Reference for Geographic Context

PubMed Query	N
Cognition	995281
Spatial Analysis	579429
Air Pollution	139478
Location	22572
Air Pollution & Cognition	2581
Location & Air Pollution & Cognition	180
Spatial Analysis & Air Pollution & Cognition	150
Location & Spatial Analysis & Air Pollution & Cognition	36

Table S.1 Number of results for topical PubMed queries and their conjunctions as of 15 September 2025. Please refer to Tables S.2 – S.8 for the actual search terms.

Term	Term cont.	Term cont.
across areas	geospatially	spatial kernels
across space	geostatistics	spatial variable
autocorrelation	in space	spatial variables
autocorrelations	kriging	spatially
by area	point pattern	spatially auto-regressive
conditional auto-regressive	point patterns	spatially autoregressive
conditional autoregressive	random field	spatially varying
disease mapping	random fields	spatio-temporally
gaussian process	spatial auto-regressive	spatiotemporally
gaussian processes	spatial autoregression	topographically
generalised additive	spatial autoregressive	topographies
generalized additive	spatial covariance	topography
geo-spatially	spatial error	topologically
geoadditive	spatial errors	topologies
geographically	spatial kernel	topology

Table S.2 Spatial query, part 1: simple search terms.

Prefix	Term	Term cont.	Term cont.
areal	analyses	epidemiology	raster
geo-spatial	analysis	factor	rasters
geospatial	approach	factors	region
geographic	approaches	feature	regions
geographical	area	features	regression
geostatistic	areas	grid	regressions
geostatistical	association	grids	relation
regional	associations	location	relations
spatial	cluster	locations	relationship
spatio-temporal	clustering	map	relationships
spatiotemporal	clusterings	mapping	scale
space-time	clusters	mappings	scales
spatial and temporal	correlation	maps	statistic
topological	correlations	measure	statistics
topographical	covariance structure	measures	structure
	covariance structures	model	structures
	data	modeling	studies
	de-confounding	modelling	study
	determinant	models	trend
	determinants	pattern	trends
	disparities	patterns	variability
	disparity	process	variable
	dispersion	processes	variables
	distribution	quantitative studies	variance
	distributions	quantitative study	variances
	effect	quantitive studies	variation
	effects	quantitive study	variations

Table S.3 Spatial query, part 2: combined search terms. The full terms are derived by forming all combinations of a prefix with a term.

Term	Term cont.	Term cont.
alzheimer	degenerative disease	neuro degenerative
brain function	degenerative diseases	neuro-degenerative
cognition	degenerative diseases	neurodegenerative
cognitive	dementia	reaction time
degenerative disease	dementias	reaction times

Table S.4 Cognition query: search terms.

Term	Term cont.
by address	LSOA
by location	LSOAs
census area	MSOA
census areas	MSOAs
census tract	postcode
census tracts	postcodes
location histories	zip code
location history	zip codes

Table S.5 Location query, part 1: simple search terms.

Prefix	Term
home	address
participant	addresses
participant's	area
participants'	areas
residential	location
	locations
	region
	regions
	ward
	wards

Table S.6 Location query, part 2: combined search terms. The full terms are derived by forming all combinations of a prefix with a term.

Term	Term cont.	Term cont.
air contaminant	fine particles	no(x)
air contaminants	nitrogen oxide	pm1
air pollutant	nitrogen oxides	pm(1)
air pollutants	nitrogen dioxide	pm2.5
air pollution	nitrogen dioxides	pm(2.5)
air quality	particulate matter	pm10
atmospheric contaminant	ultrafine particle	pm(10)
atmospheric contaminants	ultrafine particles	ufp
atmospheric pollutant	no2	ufps
atmospheric pollutants	no(2)	
fine particle	nox	

Table S.7 Pollution query, part 1: simple search terms.

Term	Term cont.	Term cont.
ammonia exposure	exposure to o(3)pb	o(3)pb exposures
ammonia exposures	exposure to o3	o3 exposure
benzene exposure	exposure to ozone	o3 exposures
benzene exposures	exposure to so2	ozone exposure
carbon monoxide exposure	exposure to sulfur dioxide	ozone exposures
carbon monoxide exposures	exposure to voc	so2 exposure
dioxin exposure	exposure to volatile organic compound	so2 exposures
dioxin exposures	exposure to volatile organic compounds	sulfur dioxide exposure
exposure to ammonia	lead exposure	sulfur dioxide exposures
exposure to benzene	lead exposures	voc exposure
exposure to carbon monoxide	mercury exposure	voc exposures
exposure to dioxin	mercury exposures	volatile organic compound exposure
exposure to lead	nh3 exposure	volatile organic compound exposures
exposure to mercury	nh3 exposures	volatile organic compounds exposure
exposure to nh3	o(3)pb exposure	volatile organic compounds exposures

Table S.8 Pollution query, part 2: exposure terms query. This query matched if there was a match of "air" or "airborne" and a match of any one of the above terms.

DOI	Year	Title
10.1038/s41598-018-30568-6	2018	Cross-sectional and longitudinal analyses of outdoor air pollution exposure and cognitive function in UK Biobank
10.1016/j.neuro.2019.06.005	2019	Association between exposure to air pollution and hippocampal volume in adults in the UK Biobank
10.1016/j.envres.2020.109365	2020	Association between exposure to air pollution and prefrontal cortical volume in adults: A cross-sectional study from the UK biobank
10.1371/journal.pone.0230829	2020	Association between exposure to air pollution and thalamus volume in adults: A cross-sectional study
10.3390/brainsci10030164	2020	Association between Exposure to Air Pollution and Total Gray Matter and Total White Matter Volumes in Adults: A Cross-Sectional Study
10.1249/MSS.0000000000002888	2022	Association of Physical Activity with Incidence of Dementia Is Attenuated by Air Pollution
10.1016/j.envres.2022.112895	2022	Exposure to air pollution and risk of incident dementia in the UK Biobank
10.1371/journal.pone.0275309	2022	Risk factors, ethnicity and dementia: A UK Biobank prospective cohort study of White, South Asian and Black participants
10.1016/j.eclinm.2023.102368	2023	Ambient air pollution and the dynamic transitions of stroke and dementia: a population-based cohort study
10.1093/aje/kwac188	2023	Associations of Air Pollution and Genetic Risk With Incident Dementia: A Prospective Cohort Study
10.3389/fnins.2023.1216686	2023	Associations of air pollution with all-cause dementia, Alzheimer's disease, and vascular dementia: a prospective cohort study based on 437,932 participants from the UK biobank
10.1007/s11356-023-31047-w	2023	Exploring the association between air pollution and Parkinson's disease or Alzheimer's disease: a Mendelian randomization study
10.34133/hds.0091	2023	Relationships of Residential Distance to Major Traffic Roads with Dementia Incidence and Brain Structure Measures: Mediation Role of Air Pollution
10.1016/j.envres.2022.114703	2023	Residential greenness and risk of incident dementia: A prospective study of 375,342 participants
10.1016/j.scitotenv.2024.173341	2024	Association between residential greenness and incident delirium: A prospective cohort study in the UK Biobank
10.1093/gerona/glae139	2024	Association of Exposure to Ambient Air Pollutants with Cognitive Performance and Dementia Risk and the Mediating Role of Pulmonary Function: Evidence From the UK Biobank
10.1186/s12889-024-17702-y	2024	Association of long-term exposure to various ambient air pollutants, lifestyle, and genetic predisposition with incident cognitive impairment and dementia
10.1016/j.envint.2024.108870	2024	Associations of ambient air pollution exposure and lifestyle factors with incident dementia in the elderly: A prospective study in the UK Biobank
10.5114/aoms/185360	2024	Causal associations of ambient particulate matter 10 and Alzheimer's disease: result from a two-sample multivariable Mendelian randomization study
10.1016/j.ecoenv.2024.116960	2024	Causal relationships between genetically predicted particulate air pollutants and neurodegenerative diseases: A two-sample Mendelian randomization study
10.3389/ijph.2024.1606868	2024	Joint Exposure to Multiple Air Pollutants, Genetic Susceptibility, and Incident Dementia: A Prospective Analysis in the UK Biobank Cohort
10.1186/s12883-024-03557-8	2024	Leading determinants of incident dementia among individuals with and without the apolipoprotein E ε4 genotype: a retrospective cohort study
10.1016/j.jhazmat.2024.136498	2024	Metabolomic profiling identifies signatures and biomarkers linking air pollution to dementia risk: A prospective cohort study
10.1016/j.ecoenv.2024.117355	2024	Residential blue space, cognitive function, and the role of air pollution in middle-aged and older adults: A cross-sectional study based on UK biobank
10.3389/frdem.2024.1402091	2024	Traffic-related air pollution and APOE4 can synergistically affect hippocampal volume in older women: new findings from UK Biobank
10.1016/j.envint.2024.109219	2024	Longitudinal associations between air pollution and incident dementia as mediated by MRI-measured brain volumes in the UK Biobank
10.1038/s41598-024-83607-w	2025	Association between air pollution and lifestyle with the risk of developing mild cognitive impairment and dementia in individuals with cardiometabolic diseases
10.1002/gps.70090	2025	The Association of Ambient Air Pollution With Dynamic Transitions of Cataract and Dementia: A UK Biobank Study
10.1111/head.15037	2025	Ambient nitrogen dioxide, temperature exposure, and migraine incidence: A large prospective cohort study

Table S.9 Previous studies of UK Biobank data involving air pollution and cognition

DOI	Year	Title
10.1016/j.envint.2021.106698	2021	Commute patterns, residential traffic-related air pollution, and lung cancer risk in the prospective UK Biobank cohort study
10.1016/j.envpol.2020.115859	2021	Links between air pollution and COVID-19 in England
10.1016/j.chemosphere.2023.139470	2023	Association between long-term exposure to low level air pollutants and incident end-stage kidney disease in the UK Biobank: A prospective cohort
10.1016/j.psychres.2023.115396	2023	Long-term exposure to ambient air pollution, genetic susceptibility, and the incidence of bipolar disorder: A prospective cohort study
10.1016/j.ecoenv.2023.115829	2024	Air pollution and the risk of incident chronic kidney disease in patients with diabetes: An exposure-response analysis
10.1016/j.jhazmat.2024.134861	2024	Estimating neighborhood-based mortality risk associated with air pollution: A prospective study
10.1016/j.scitotenv.2024.173833	2024	Greenspace and risk of obesity-related cancer in the UK Biobank cohort: An analysis of private residential gardens and other greenspace types
10.1038/s41467-024-49283-0	2024	Heterogeneous associations of multiplexed environmental factors and multidimensional aging metrics
10.1161/STROKEAHA.123.044935	2024	Joint Exposure to Ambient Air Pollutants, Genetic Risk, and Ischemic Stroke: A Prospective Analysis in UK Biobank
10.1038/s41370-023-00635-w	2024	Reconstructing individual-level exposures in cohort analyses of environmental risks: an example with the UK Biobank
10.1038/s41598-025-00921-7	2025	Impact of gene-environment interactions on atrial fibrillation and cardiac structure

Table S.10 Previous studies of UK Biobank data involving air pollution and incorporating a spatial aspect

Name	Year	~ Size	Region	Location	Cognitive Assessment Summary	URL, DOI
UK Biobank	2006	500,000	United Kingdom	Home location at 100m and 1km grid resolutions.	Touchscreen assessments of reaction time, memory, and fluid intelligence. A subset received more comprehensive testing during imaging.	https://www.ukbiobank.ac.uk/<br><br>10.1371/journal.pmed.1001779
English Longitudinal Study of Ageing (ELSA)	2002	12,000	England	Local Authority Districts for Waves 1–5, LSOAs for Waves 6–10.	Includes cognitive function measures. A sub-study utilized the Harmonized Cognitive Assessment Protocol (HCAP).	https://www.elsa-project.ac.uk/<br><br>10.1093/ije/dys168
Understanding Society: The UK Household Longitudinal Study	2009	100,000	United Kingdom	Home location at LSOA-level or national grid (1m).	Includes memory and number tasks to track mental functioning across the lifespan.	https://www.understandingsociety.ac.uk/<br><br>10.14301/llcs.v3i1.159
Generation Scotland	2006	40,000	Scotland	Optional linkage to other records.	Assesses multiple cognitive domains, including verbal declarative memory, executive function, and processing speed.	https://genscot.ed.ac.uk/<br><br>10.1136/bmjopen-2024-084719
Avon Longitudinal Study of Parents and Children (ALSPAC)	1991	14,000	Avon, South West England	Residential postcode information is collected throughout.	Cognitive data collected throughout participants' lives, including the WISC and verbal fluency tests.	http://www.bristol.ac.uk/alspac/<br><br>10.1093/ije/dyz063
1958 National Child Development Study (NCDS)	1958	17,000	Great Britain	LSOAs of residential postcode.	Cognitive ability and function tracked across the life course, including verbal ability in childhood and literacy/numeracy later.	https://cls.ucl.ac.uk/cls-studies/1958-national-child-development-study/<br><br>10.1177/003591577306600749
1970 British Cohort Study (BCS70)	1970	17,000	Great Britain	LSOAs of residential postcode.	Cognitive assessments covering literacy, numeracy, and verbal and non-verbal skills.	https://cls.ucl.ac.uk/cls-studies/1970-british-cohort-study/<br>10.1093/ije/dyl174

Table S.11 Cohorts identified for the United Kingdom that contain cognitive and residential information.

Variable	Field Number	Field Description	Comment
Easting	22702	Home location - east co-ordinate	Not entered in any analysis as a covariate
Northing	22704	Home location - north co-ordinate	Not entered in any analysis as a covariate
Reaction time	20023	Mean time to correctly identify matches	–
Completion time	400	Time to complete round	Array index 0 for round 1
Sex	31	Sex	–
Age	21003	Age when attended assessment centre	–
Is ethnic minority	21000	Ethnic background	True if coding not in {1001, 1002, 1003}
Formal qualifications	6138	Qualifications	Number of coded qualifications: 0...6
Basal metabolic rate	23105	Basal metabolic rate	–
Subjective health score	2178	Overall health rating	Recoded as 0 to 3 with increasing health
Alcohol consumption level	1558	Alcohol intake frequency	Recoded as 0 to 6 with increasing frequency
Anxious feelings	1980	Anxious feelings	–
Walking pace score	924	Usual walking pace	Recoded as 0 to 2 from "slow" to "brisk".
Freq. of social visits	1031	Frequency of friend/family visits	Recoded as 0 to 6 from "no friends/family" to "daily"
Greenspace %	24500	Greenspace %, buffer 1000m	–
NO_2	24003	Nitrogen dioxide air pollution; 2010	–
NO_x	24004	Nitrogen oxides air pollution; 2010	–
$PM_{2.5}$	24006	Particulate matter air pollution (pm2.5); 2010	–

Table S.12 Variables derived from UK Biobank.

	Birmingham	Leeds	Liverpool	Manchester
Number of participants	17829	19161	15830	19601
Reaction time (Mean ± SD) [ms]	566.16 ± 107.09	547.49 ± 96.96	564.72 ± 104.67	553.12 ± 98.96
Female	571.26 ± 105.79	553.90 ± 97.64	570.26 ± 104.06	558.30 ± 98.98
Male	560.28 ± 108.29	539.28 ± 95.47	557.73 ± 105.02	547.17 ± 98.61
Completion time (Mean ± SD) [ds]	111.55 ± 69.64	112.41 ± 81.45	119.33 ± 88.29	124.13 ± 104.33
Female	110.87 ± 67.14	114.45 ± 83.91	121.76 ± 89.73	126.74 ± 108.02
Male	112.34 ± 72.43	109.80 ± 78.12	116.27 ± 86.35	121.12 ± 99.85
Sex (%)				
Female	9554 (53.6)	10760 (56.2)	8837 (55.8)	10474 (53.4)
Male	8275 (46.4)	8401 (43.8)	6993 (44.2)	9127 (46.6)
Age (Mean ± SD) [years]	56.40 ± 8.14	56.10 ± 7.92	56.81 ± 7.85	55.91 ± 8.13
Female	56.04 ± 8.05	55.84 ± 7.81	56.52 ± 7.79	55.73 ± 8.04
Male	56.83 ± 8.22	56.43 ± 8.04	57.19 ± 7.90	56.11 ± 8.24
Ethnic minority (%)	1724 (9.7)	559 (2.9)	371 (2.3)	1022 (5.2)
Female	919 (9.6)	285 (2.6)	210 (2.4)	508 (4.9)
Male	805 (9.7)	274 (3.3)	161 (2.3)	514 (5.6)
Formal qualifications (Mean ± SD) [0…6]	1.67 ± 1.31	1.64 ± 1.31	1.58 ± 1.30	1.57 ± 1.31
Female	1.67 ± 1.30	1.63 ± 1.32	1.59 ± 1.29	1.59 ± 1.32
Male	1.67 ± 1.32	1.65 ± 1.31	1.57 ± 1.31	1.56 ± 1.31
Basal metabolic rate (Mean ± SD) [kJ]	6632.06 ± 1283.34	6592.26 ± 1279.23	6565.12 ± 1281.62	6578.60 ± 1278.61
Female	5710.16 ± 684.00	5699.95 ± 656.34	5677.88 ± 673.28	5656.97 ± 674.15
Male	7696.44 ± 945.55	7735.13 ± 924.46	7686.31 ± 945.25	7636.25 ± 946.56
Subjective health score (Mean ± SD) [0…3]	1.79 ± 0.72	1.88 ± 0.70	1.83 ± 0.74	1.83 ± 0.74
Female	1.82 ± 0.71	1.90 ± 0.69	1.86 ± 0.73	1.86 ± 0.73
Male	1.76 ± 0.73	1.86 ± 0.71	1.80 ± 0.76	1.79 ± 0.76
Alcohol consumption level (Mean ± SD) [0…6]	2.89 ± 1.56	3.10 ± 1.47	2.93 ± 1.45	3.03 ± 1.51
Female	2.59 ± 1.55	2.85 ± 1.50	2.67 ± 1.46	2.79 ± 1.52
Male	3.22 ± 1.49	3.42 ± 1.37	3.26 ± 1.37	3.32 ± 1.45
Anxious feelings (%)	10052 (56.4)	10969 (57.2)	9195 (58.1)	11159 (56.9)
Female	6179 (64.7)	6954 (64.6)	5791 (65.5)	6737 (64.3)
Male	3873 (46.8)	4015 (47.8)	3404 (48.7)	4422 (48.4)
Walk pace score (Mean ± SD) [0…2]	1.30 ± 0.60	1.32 ± 0.60	1.25 ± 0.62	1.26 ± 0.61
Female	1.28 ± 0.61	1.31 ± 0.60	1.24 ± 0.63	1.25 ± 0.62
Male	1.32 ± 0.60	1.34 ± 0.60	1.26 ± 0.62	1.27 ± 0.61
Frequency of social visits (Mean ± SD) [0…6]	4.22 ± 1.11	4.28 ± 1.11	4.40 ± 1.12	4.32 ± 1.13
Female	4.34 ± 1.08	4.42 ± 1.07	4.54 ± 1.07	4.49 ± 1.07
Male	4.07 ± 1.13	4.09 ± 1.13	4.22 ± 1.16	4.13 ± 1.16

Table S.13 Descriptive statistics of participants by city.

	Birmingham	Leeds	Liverpool	Manchester
Greenspace % (Mean ± SD)	31.16 ± 12.99	47.71 ± 16.56	34.33 ± 13.56	36.96 ± 13.76
NO_2 (Mean ± SD) [μg / m^3]	29.98 ± 4.29	26.78 ± 5.59	29.30 ± 6.16	30.36 ± 5.03
NO_x (Mean ± SD) [μg / m^3]	45.45 ± 8.91	44.28 ± 11.66	48.93 ± 13.26	49.87 ± 11.80
$PM_{2.5}$ (Mean ± SD) [μg / m^3]	9.80 ± 0.59	10.05 ± 0.91	10.49 ± 1.00	10.44 ± 0.87

Table S.14 Descriptive statistics of environmental variables by city.

City	Reaction Time x ...	Conclusive Regions	Inconclusive Regions
Birmingham	NO_2 Pollution	++<br>#1, N=155, C=5: *Attwood Green, Edgbaston, Park Central*<br>--<br>#2, N=229, C=3: *Boldmere, Short Heath*<br>#3, N=54, C=1: *Little Sutton*	–
	NO_x Pollution	++<br>#4, N=55, C=1: *Park Central*<br>--<br>#5, N=73, C=1	–
	$PM_{2.5}$ Pollution	++<br>#6, N=136, C=4: *Attwood Green, Edgbaston, Park Central*	–
Leeds	NO_2 Pollution	--<br>#1, N=52, C=2<br>++<br>#2, N=52, C=4: *Little London, Lovell Park, Woodhouse*	–
	NO_x Pollution	++<br>#3, N=40, C=3: *Little London, Woodhouse*	–
	$PM_{2.5}$ Pollution	-+<br>#4, N=19, C=2<br>++<br>#5, N=138, C=6: *Chapeltown, Little London, Lovell Park, Woodhouse*	–
Liverpool	NO_2 Pollution	+-<br>#1, N=25, C=1<br>++<br>#2, N=103, C=5: *Edge Hill, Everton, Georgian Quarter, Islington, Kirkdale*	–
	NO_x Pollution	++<br>#3, N=284, C=11: *Edge Hill, Everton, Georgian Quarter, Islington, Kensington*	–
	$PM_{2.5}$ Pollution	++<br>#4, N=111, C=6: *Edge Hill, Everton, Georgian Quarter, Islington, Kirkdale*<br>--<br>#5, N=73, C=1	–
Manchester	NO_2 Pollution	-+<br>#2, N=30, C=2<br>++<br>#3, N=30, C=2<br>#4, N=55, C=4: *Beswick*	#1, N=609, C=11
	NO_x Pollution	-+<br>#7, N=44, C=4	#5, N=73, C=1<br>#6, N=91, C=2
	$PM_{2.5}$ Pollution	--<br>#8, N=150, C=1<br>++<br>#10, N=12, C=2	#9, N=599, C=14: *Gorse Hill, Salford Quays, Stretford*

Table S.15 Summary of all significant areas for the interaction between reaction time and each pollution variable and city as shown in Figures 1–4 (A) in terms of region number in the figures (#), number of participants (N), number of 1 km^2 cells (C) and place labels extracted from Open Street Map data. Conclusive regions are grouped by the effect signs of cognition times and pollution, as indicated by ++, --, +- and -+.

City	Completion Time x ...	Conclusive Regions	Inconclusive Regions
Birmingham	NO_2 Pollution	++<br>#7, N=359, C=16: *Attwood Green, Digbeth, Edgbaston,*<br>*Jewellery Quarter, Ladywood*<br>-+<br>#8, N=16, C=2: *Dixons Green, Dudley, Kates Hill*	–
	NO_x Pollution	++<br>#9, N=76, C=4: *Attwood Green, Ladywood, Park Ctrl*<br>#10, N=19, C=1<br>-+<br>#11, N=59, C=4: *Dixons Green, Dudley, Kates Hill*	–
	$PM_{2.5}$ Pollution	++<br>#12, N=403, C=18: *Attwood Green, Digbeth, Edgbaston, Jewellery Quarter, Ladywood*	#13, N=202, C=11: *Dixons Green, Dudley, Kates Hill, Little Fields, Oakham*
Leeds	NO_2 Pollution	++<br>#6, N=168, C=11: *Cross Green, East End Park, Holbeck Urban Village, Hunslet, Hyde Park*	–
	NO_x Pollution	++<br>#7, N=95, C=8: *Cross Green, East End Park, Holbeck Urban Village, Hunslet, Richmond Hill*	–
	$PM_{2.5}$ Pollution	++<br>#8, N=139, C=13: *Holbeck Urban Village, Hunslet, Hyde Park, Little London, Lovell Park*	–
Liverpool	NO_2 Pollution	++<br>#6, N=214, C=7: *Edge Hill, Georgian Quarter, Islington,*<br>*Ropewalks, Vauxhall*	–
	NO_x Pollution	++<br>#7, N=193, C=5: *Edge Hill, Georgian Quarter*	–
	$PM_{2.5}$ Pollution	++<br>#8, N=346, C=11: *Edge Hill, Everton, Georgian Quarter,*<br>*Islington, Kensington*	–
Manchester	NO_2 Pollution	++<br>#12, N=318, C=6: *Longsight, Rusholme, Victoria Park*<br>#13, N=64, C=6: *Collyhurst, Strangeways*	#11, N=889, C=12: *Sale Moor, Stretford*
	NO_x Pollution	++<br>#16, N=128, C=2: *Rusholme*<br>#17, N=48, C=5: *Strangeways*<br>#18, N=50, C=2: *Bury*	#14, N=12, C=4<br>#15, N=385, C=5
	$PM_{2.5}$ Pollution	--<br>#19, N=20, C=6: *Cheadle Hulme*<br>#20, N=150, C=1<br>++<br>#21, N=63, C=1<br>#22, N=123, C=4: *Bury, Fairfield, Fishpool*	–

Table S.16 Summary of all significant areas for the interaction between completion time and each pollution variable and city as shown in Figures 1–4 (B) in terms of region number in the figures (#), number of participants (N), number of 1 km^2 cells (C) and place labels extracted from Open Street Map data. Conclusive regions are grouped by the effect signs of cognition times and pollution, as indicated by ++, --, +- and -+.

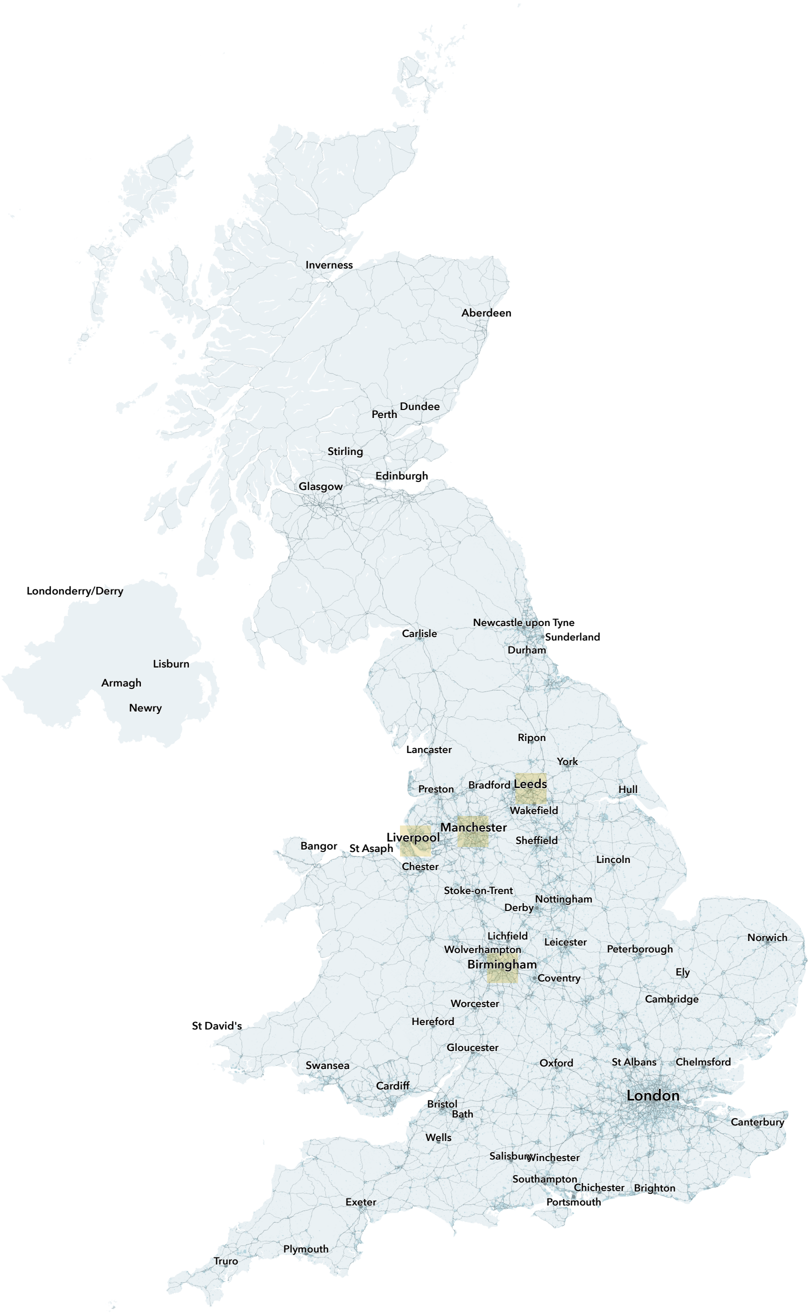

Figure S.1 Location of study regions within the United Kingdom

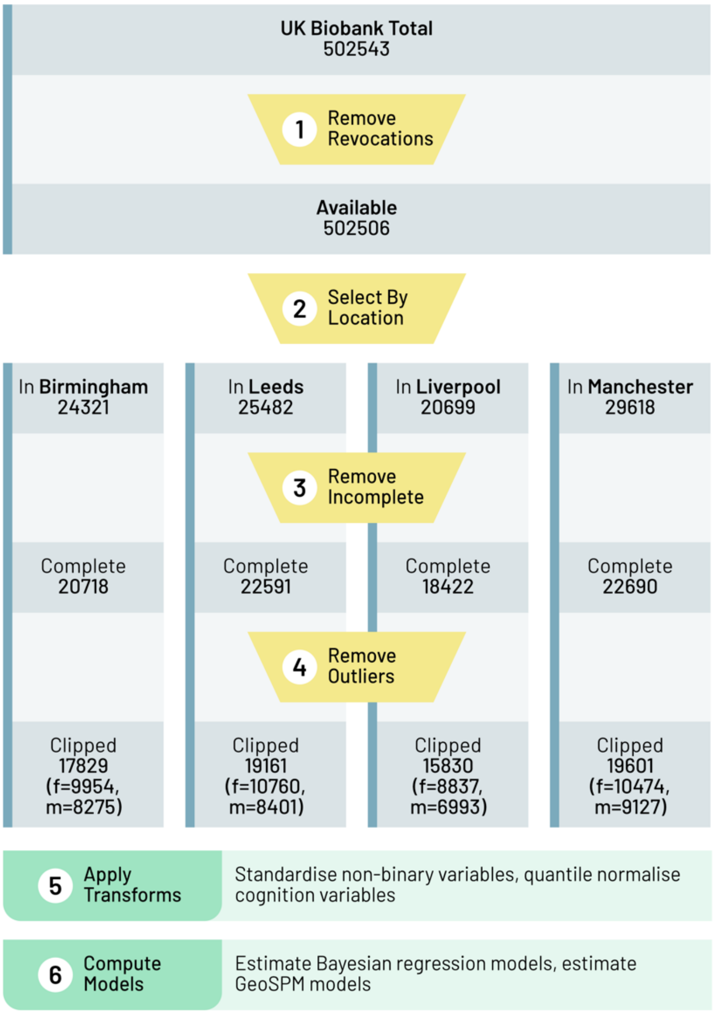

Figure S.2 Cohort selection and study design.

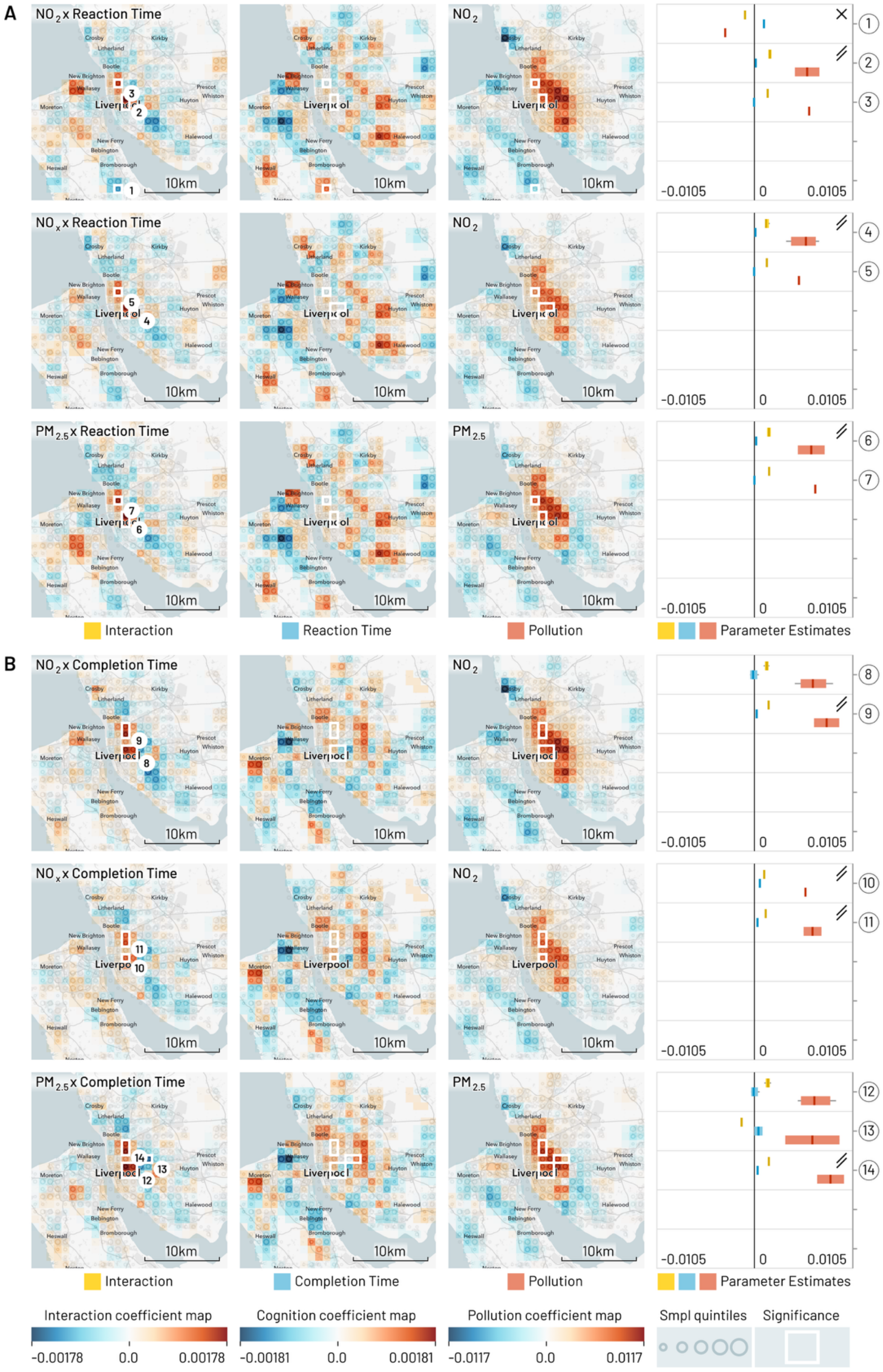

Figure S.3 Geographic regression maps showing interaction and individual effects for cognition and air pollution in Liverpool with smoothing applied (95% of kernel density within a 1.25km diameter).

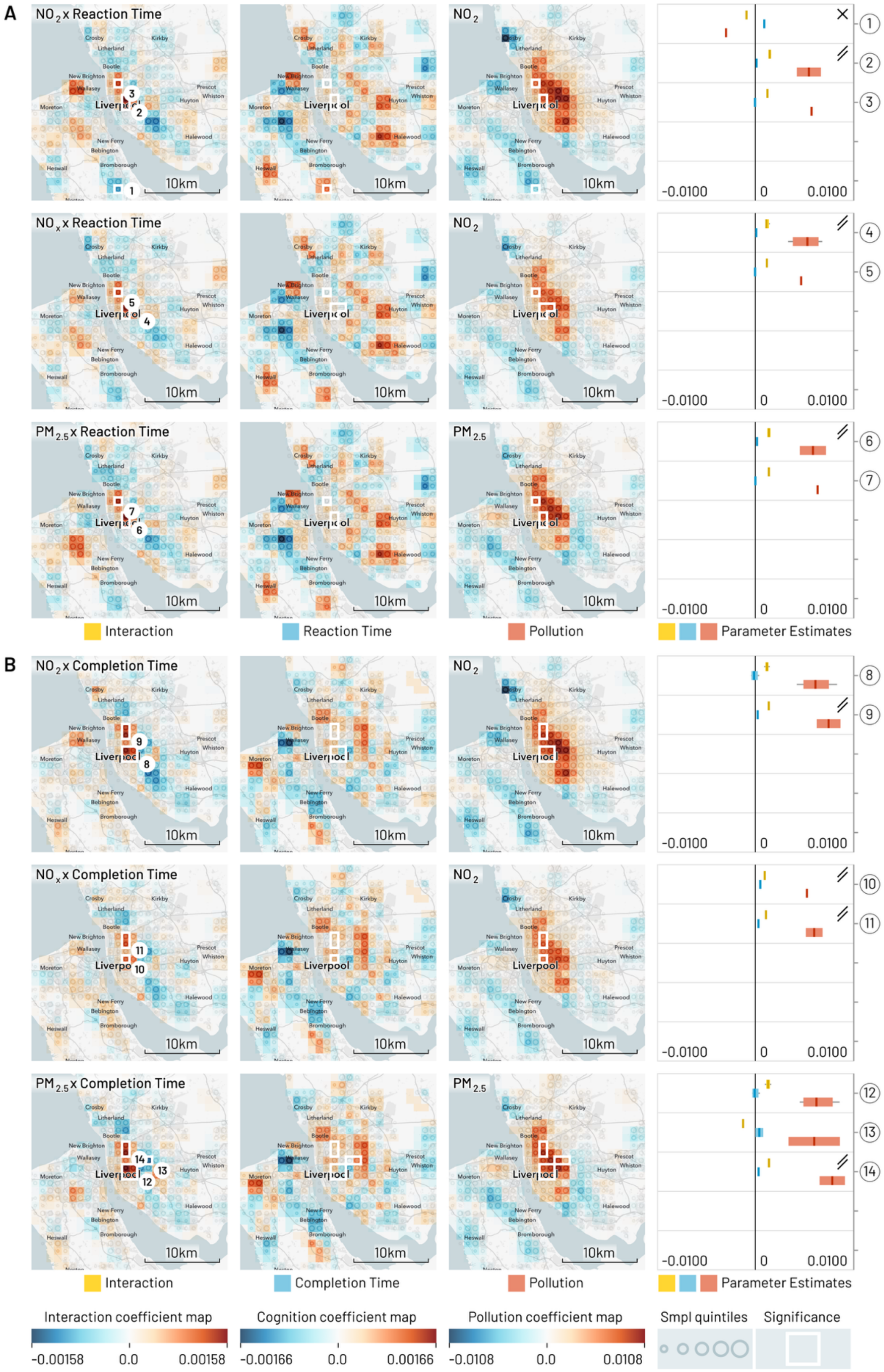

Figure S.4 Geographic regression maps showing interaction and individual effects for cognition and air pollution in Liverpool with smoothing applied (95% of kernel density within a 2.5km diameter).

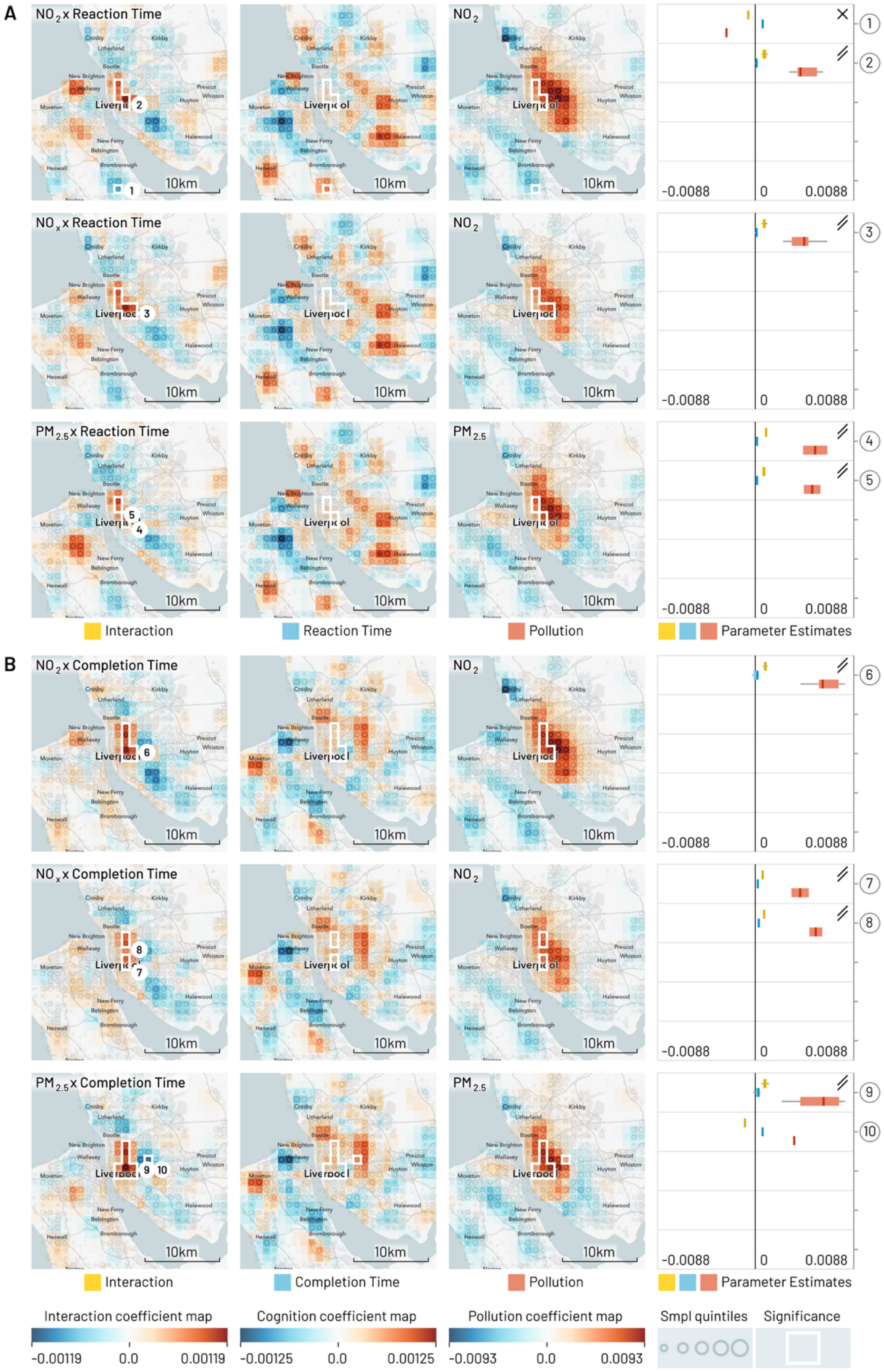

Figure S.5 Geographic regression maps showing interaction and individual effects for cognition and air pollution in Liverpool with smoothing applied (95% of kernel density within a 3.75km diameter).

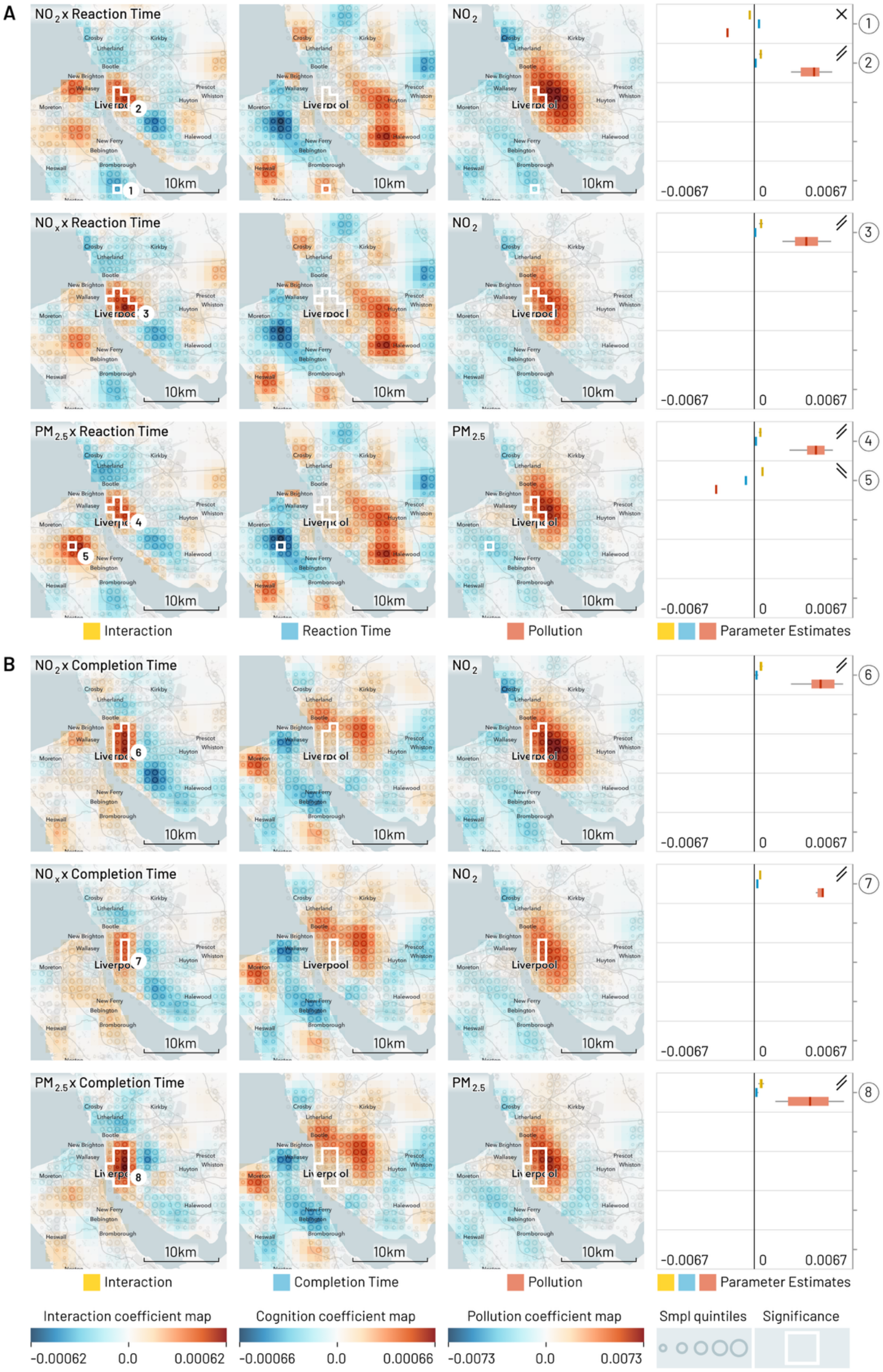

Figure S.6 Geographic regression maps showing interaction and individual effects for cognition and air pollution in Liverpool with smoothing applied (95% of kernel density within a 6.25km diameter).

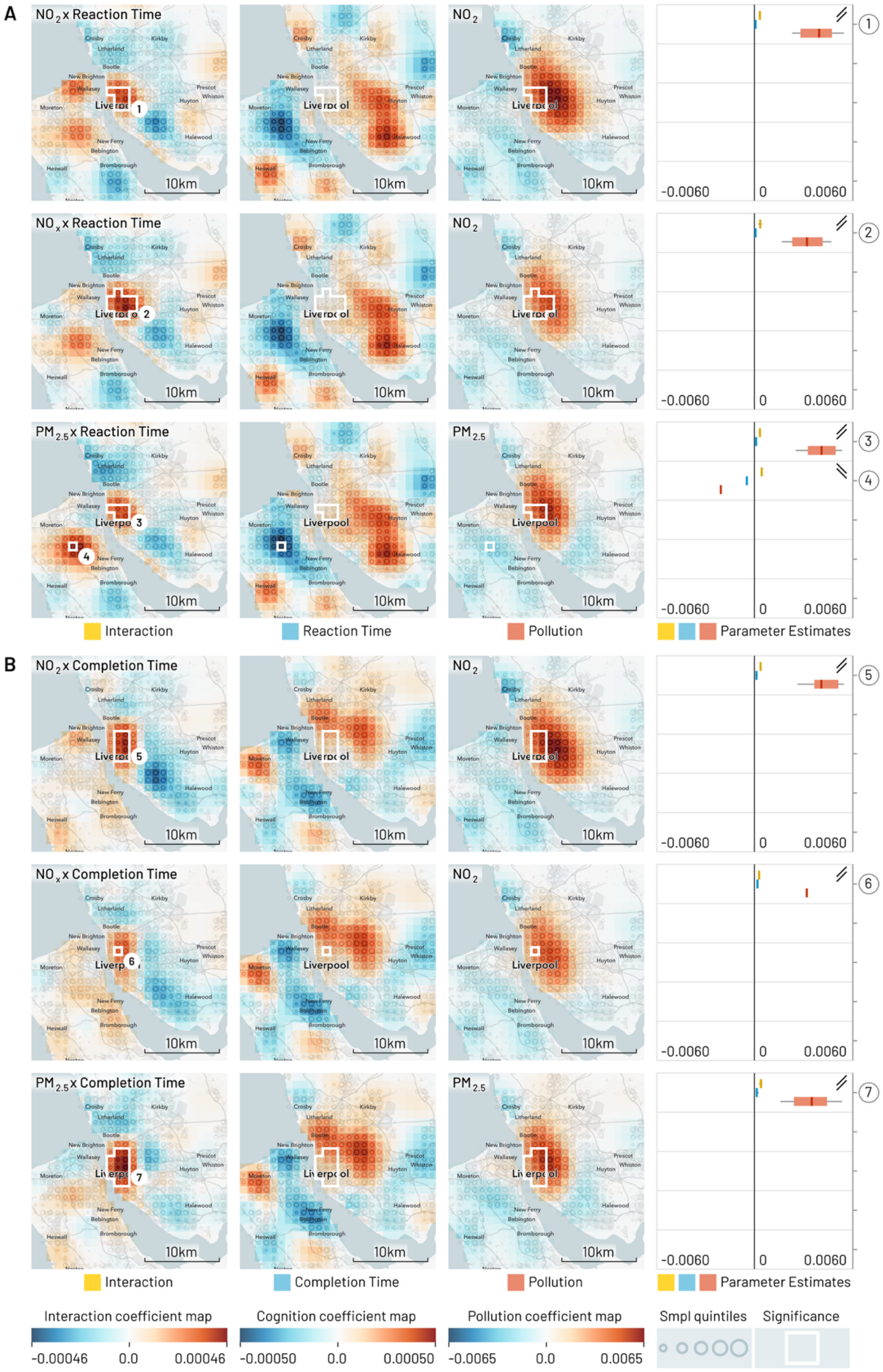

Figure S.7 Geographic regression maps showing interaction and individual effects for cognition and air pollution in Liverpool with smoothing applied (95% of kernel density within a 7.5km diameter).

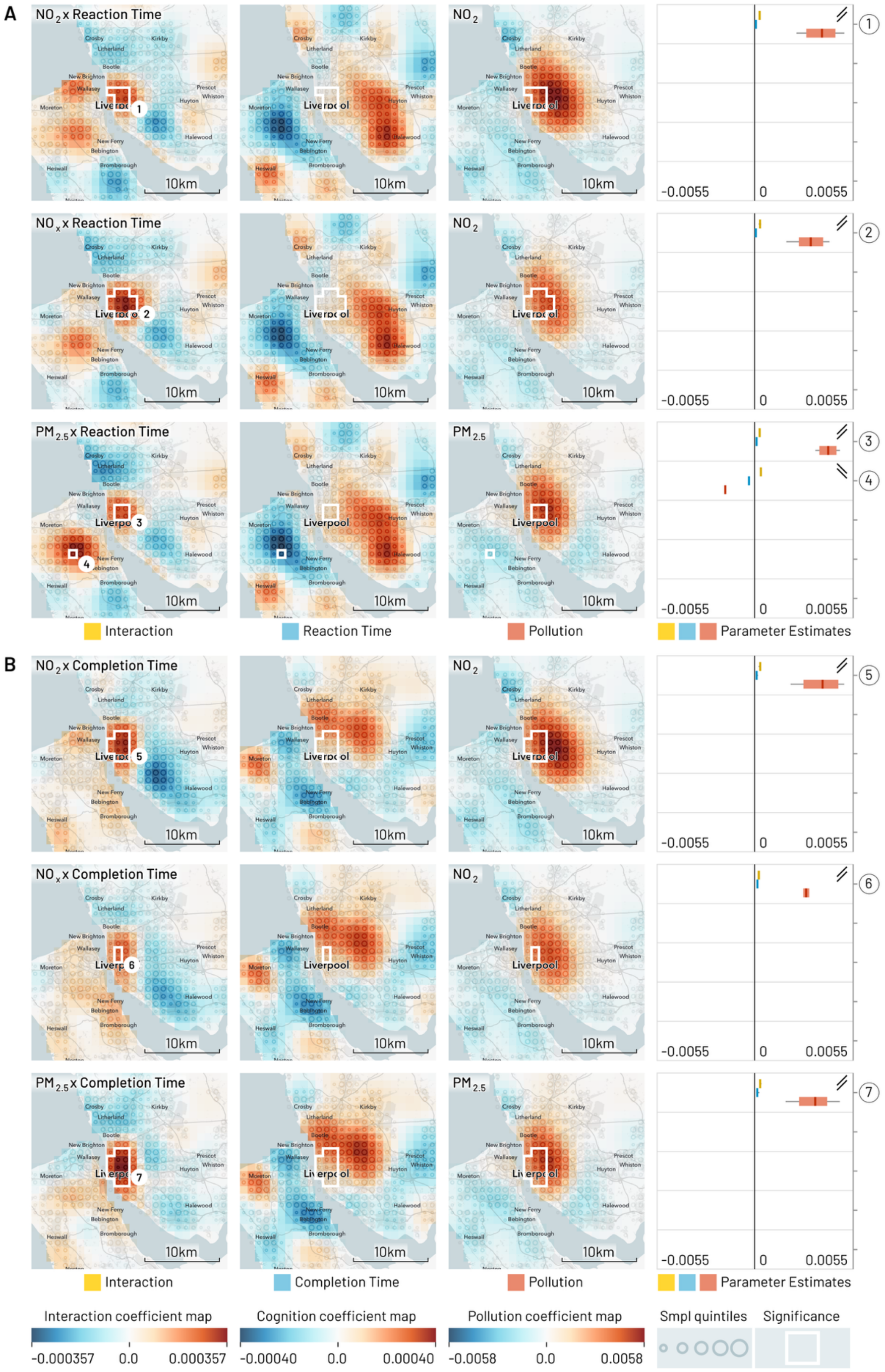

Figure S.8 Geographic regression maps showing interaction and individual effects for cognition and air pollution in Liverpool with smoothing applied (95% of kernel density within an 8.75km diameter).

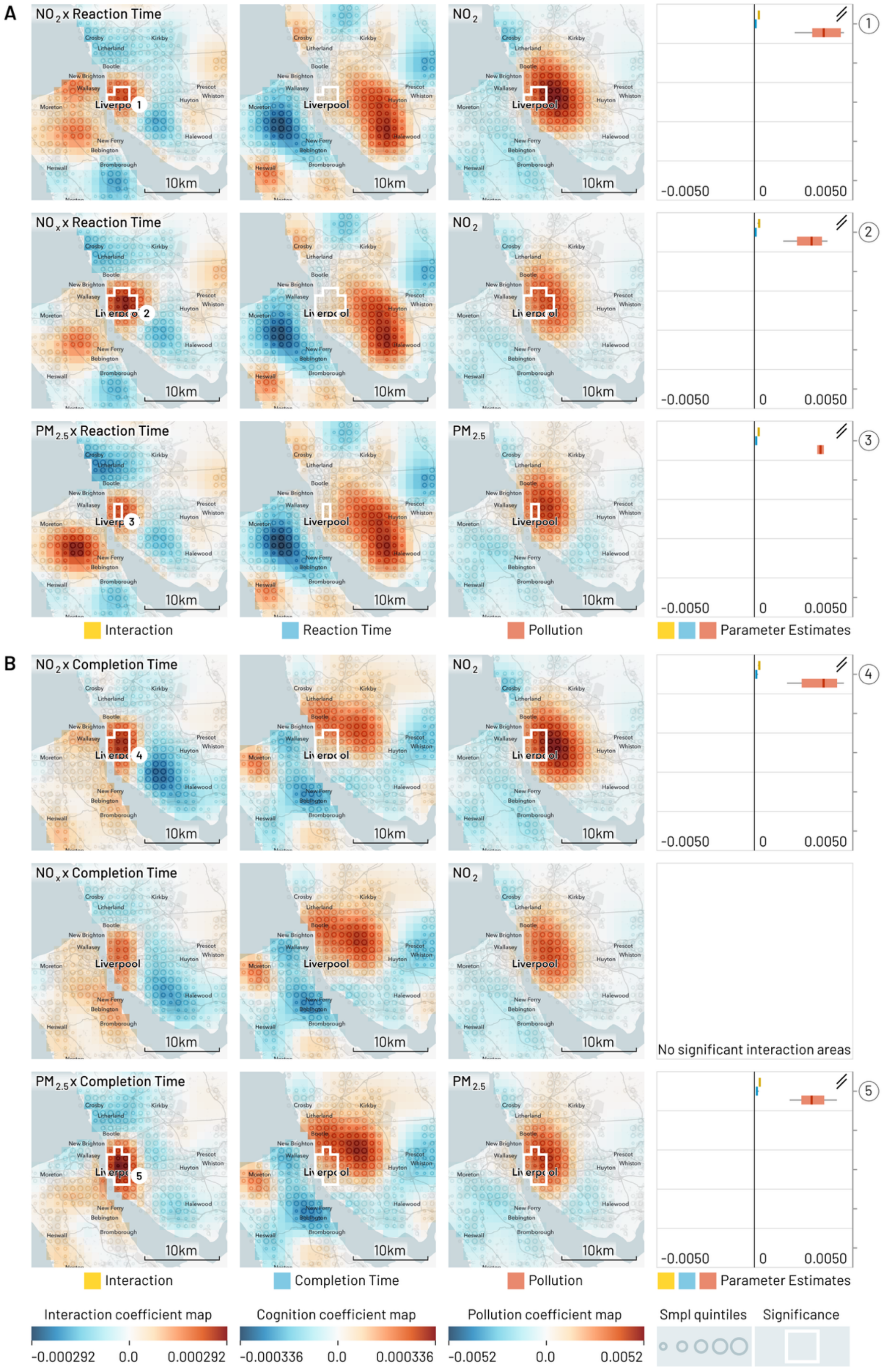

Figure S.9 Geographic regression maps showing interaction and individual effects for cognition and air pollution in Liverpool with smoothing applied (95% of kernel density within a 10km diameter).

Sensitivity Analyses: Kernel Sizes Birmingham

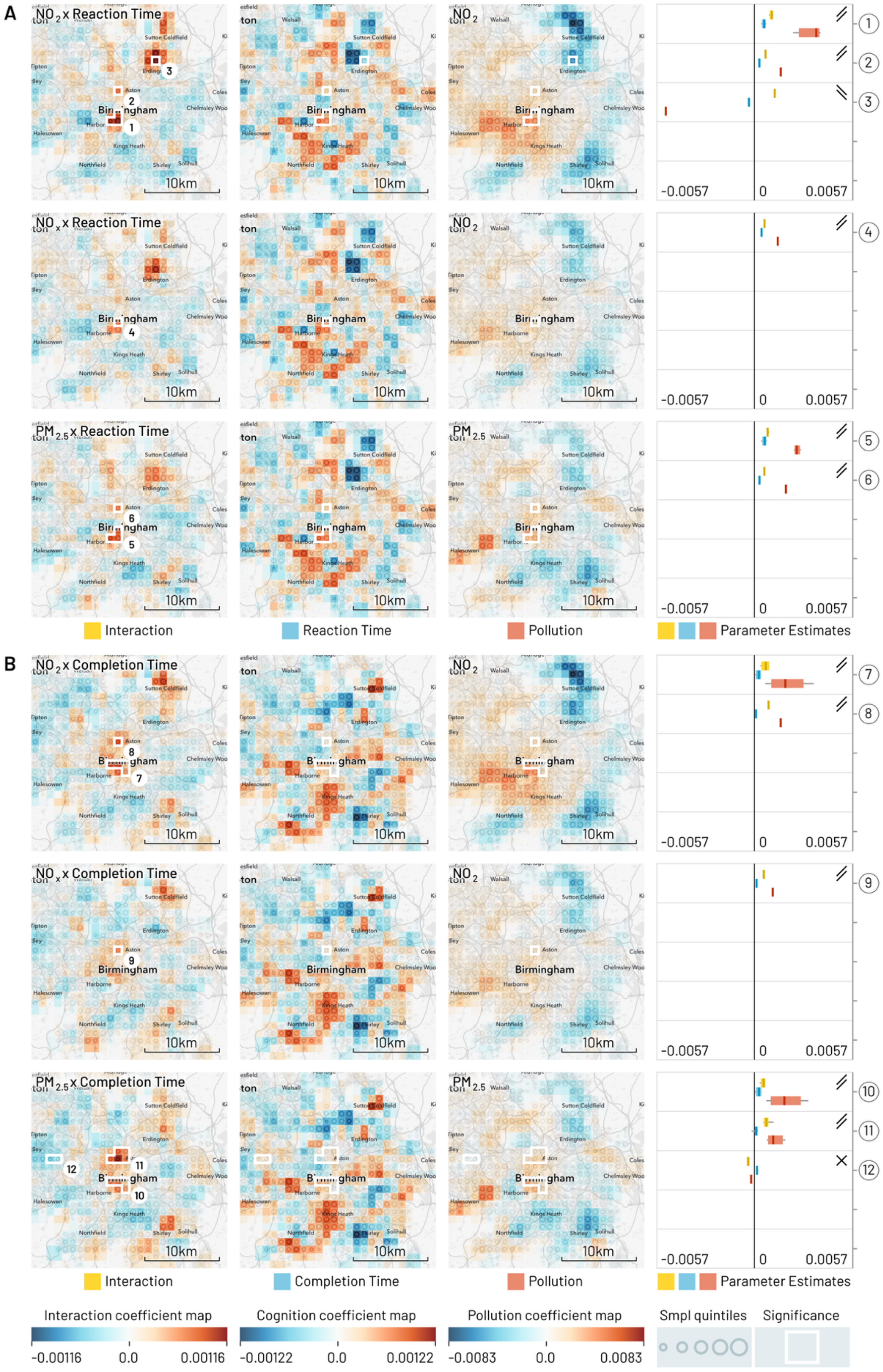

Figure S.10 Geographic regression maps showing interaction and individual effects for cognition and air pollution in Birmingham with smoothing applied (95% of kernel density within a 2.5km diameter).

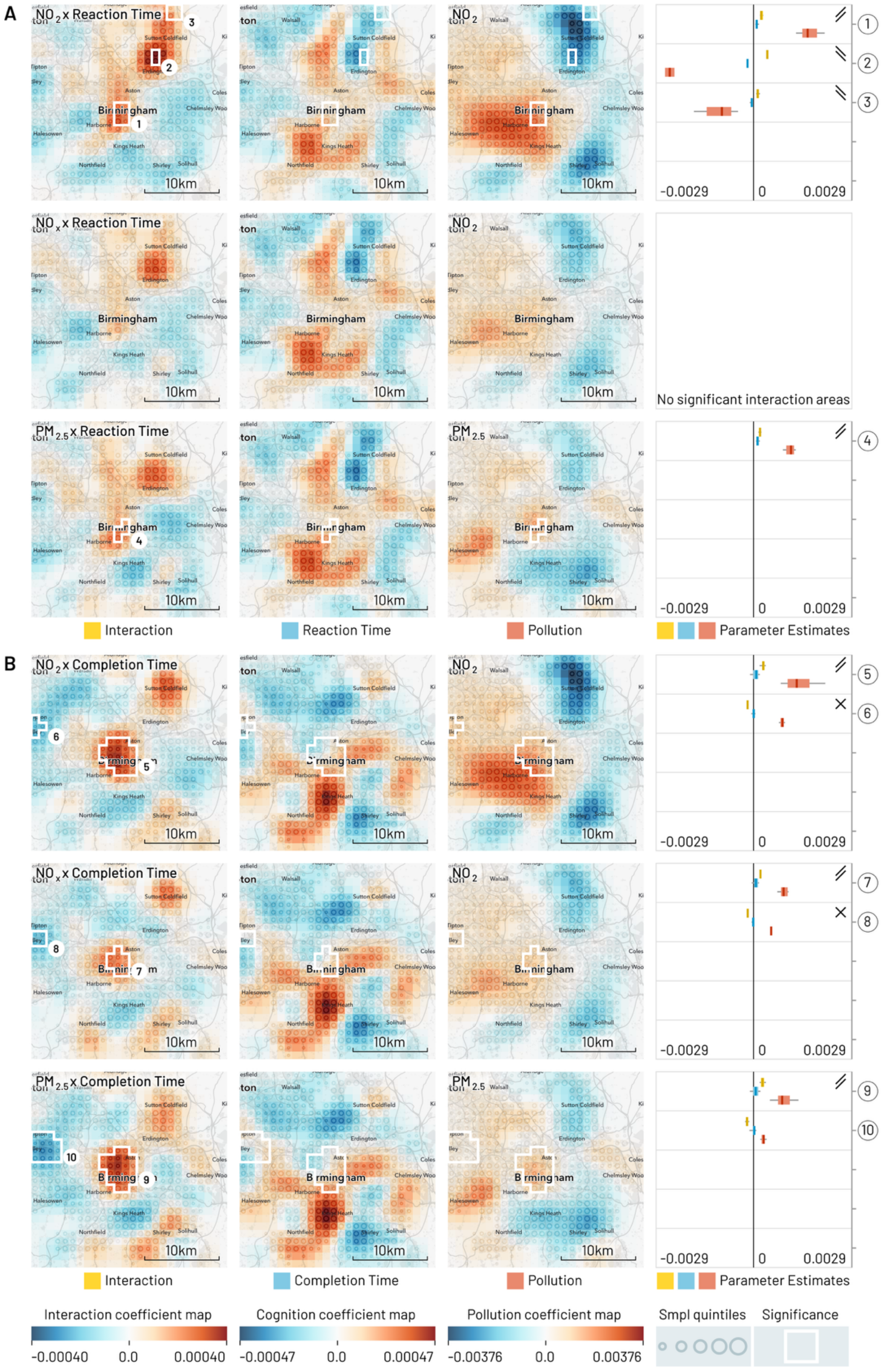

Figure S.11 Geographic regression maps showing interaction and individual effects for cognition and air pollution in Birmingham with smoothing applied (95% of kernel density within a 7.5km diameter).

Sensitivity Analyses: Kernel Sizes Leeds

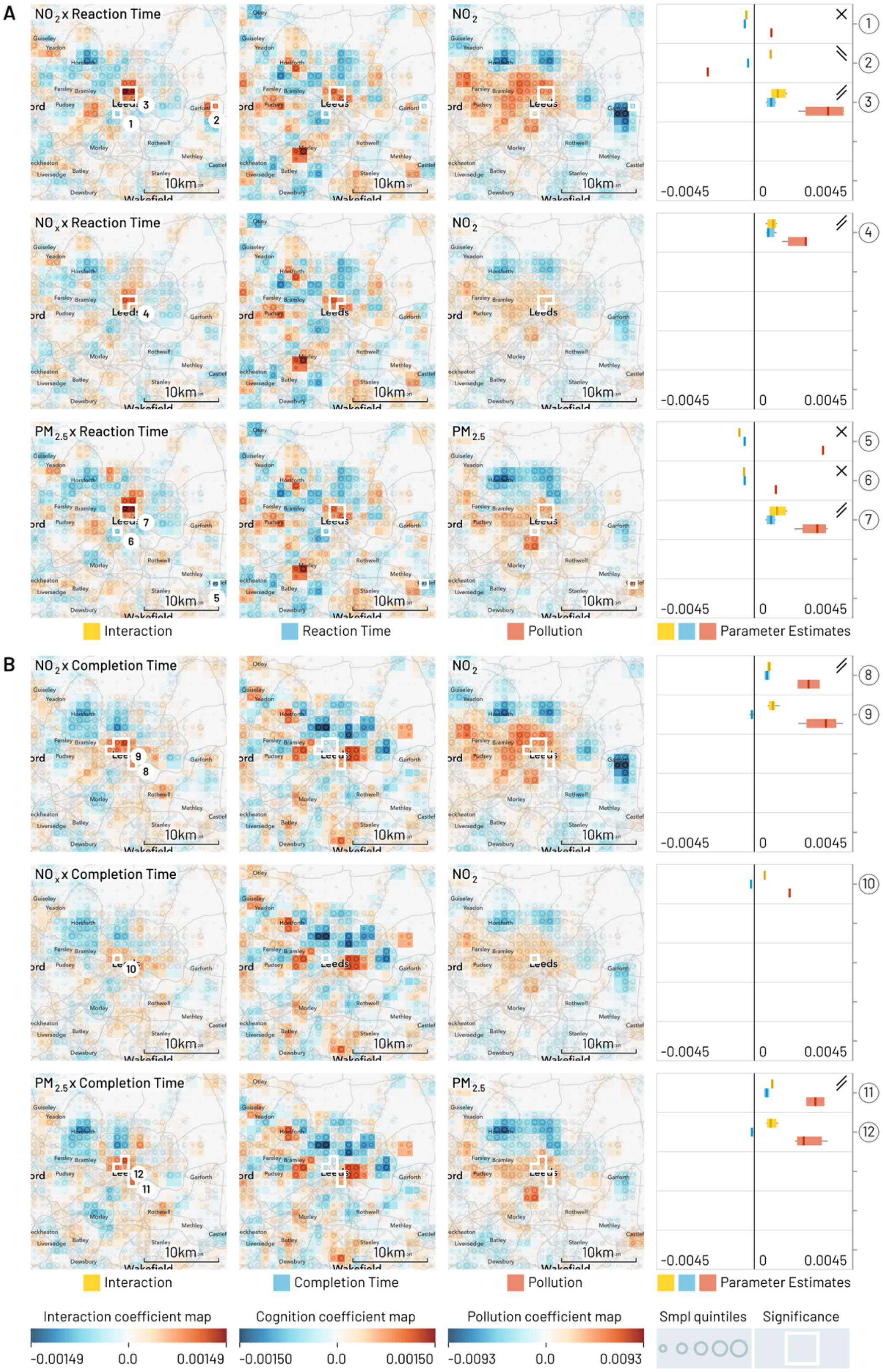

Figure S.12 Geographic regression maps showing interaction and individual effects for cognition and air pollution in Leeds with smoothing applied (95% of kernel density within a 2.5km diameter).

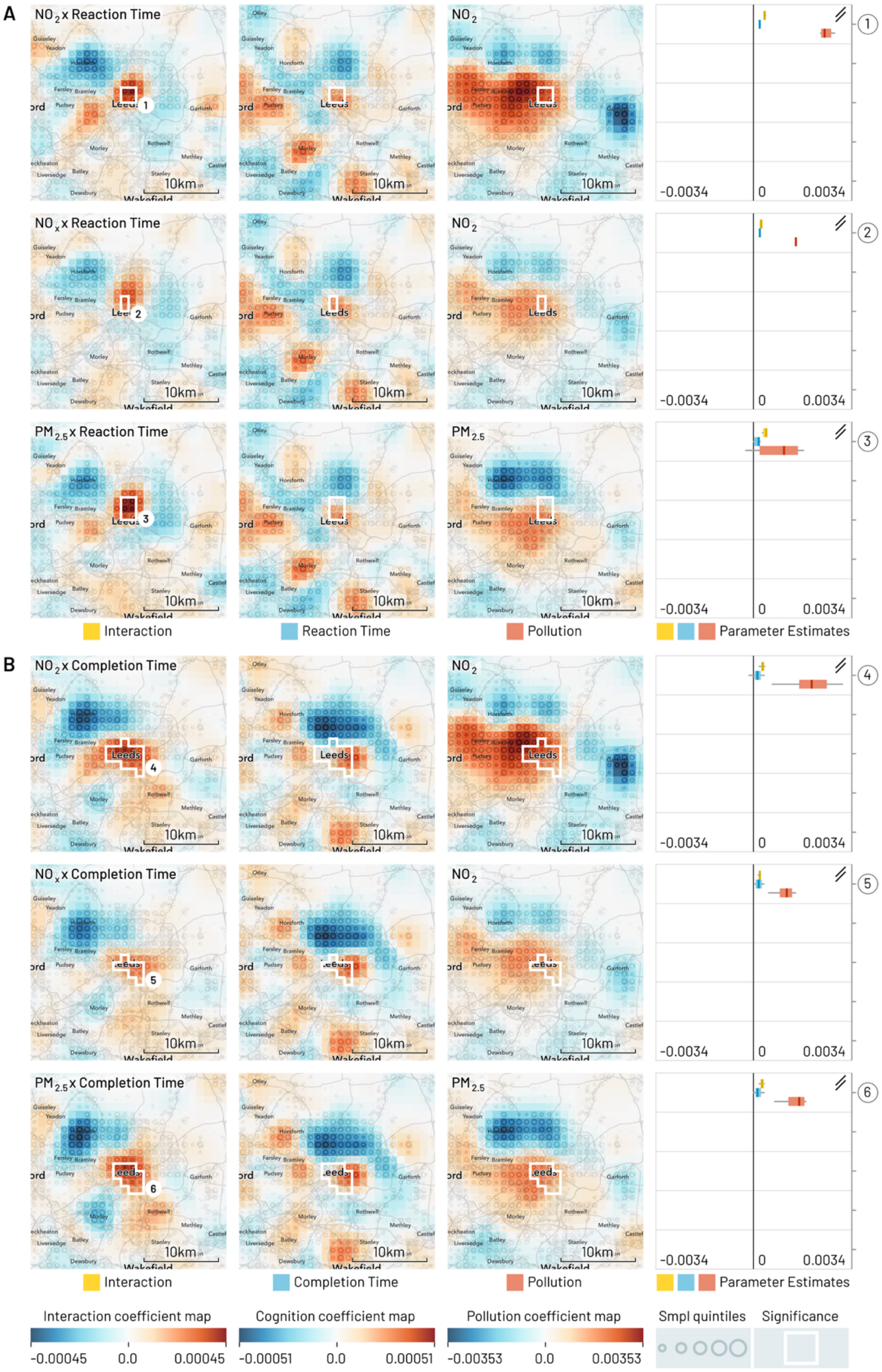

Figure S.13 Geographic regression maps showing interaction and individual effects for cognition and air pollution in Leeds with smoothing applied (95% of kernel density within a 7.5km diameter).

Sensitivity Analyses: Kernel Sizes Manchester

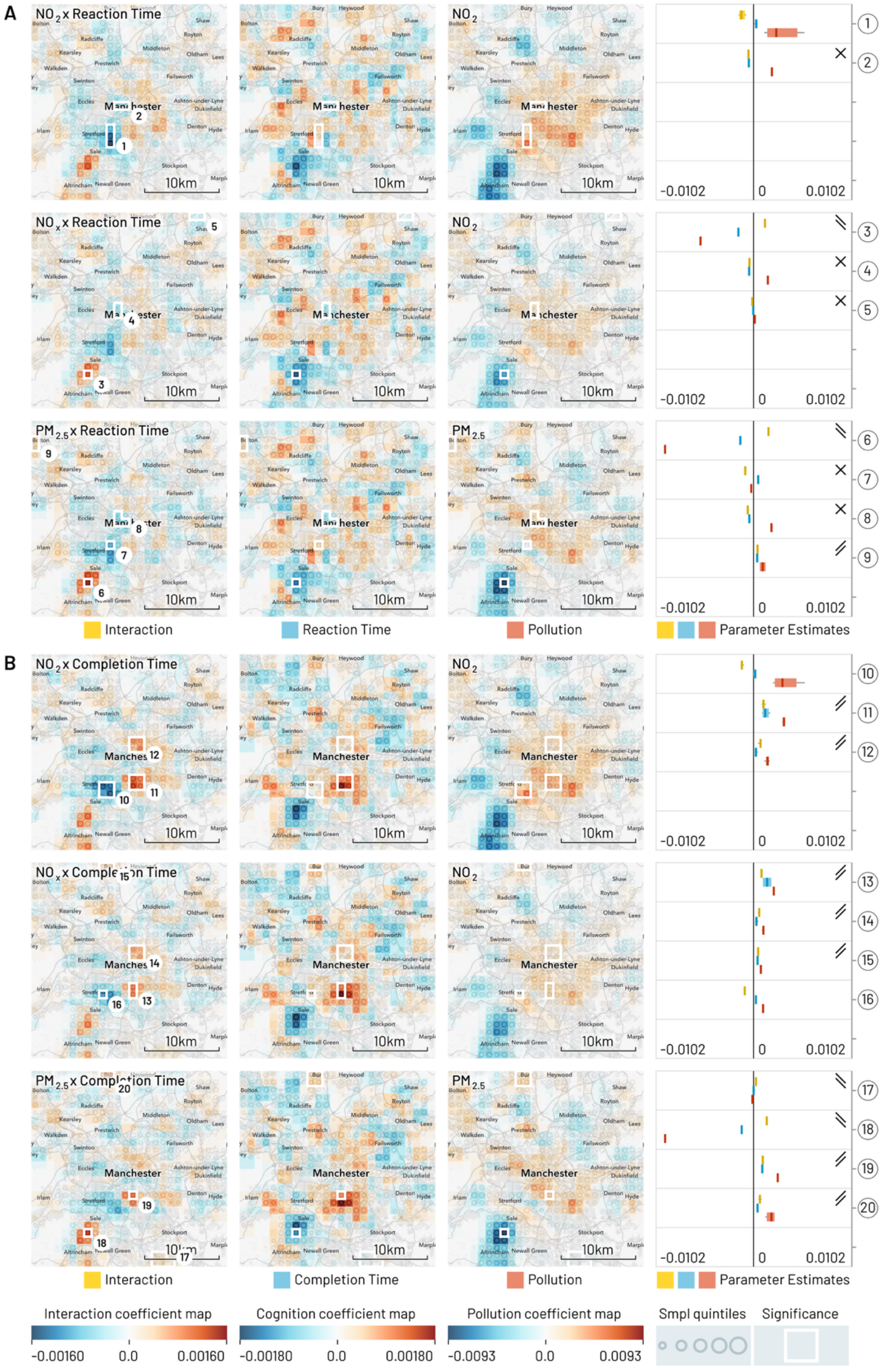

Figure S.14 Geographic regression maps showing interaction and individual effects for cognition and air pollution in Manchester with smoothing applied (95% of kernel density within a 2.5km diameter).

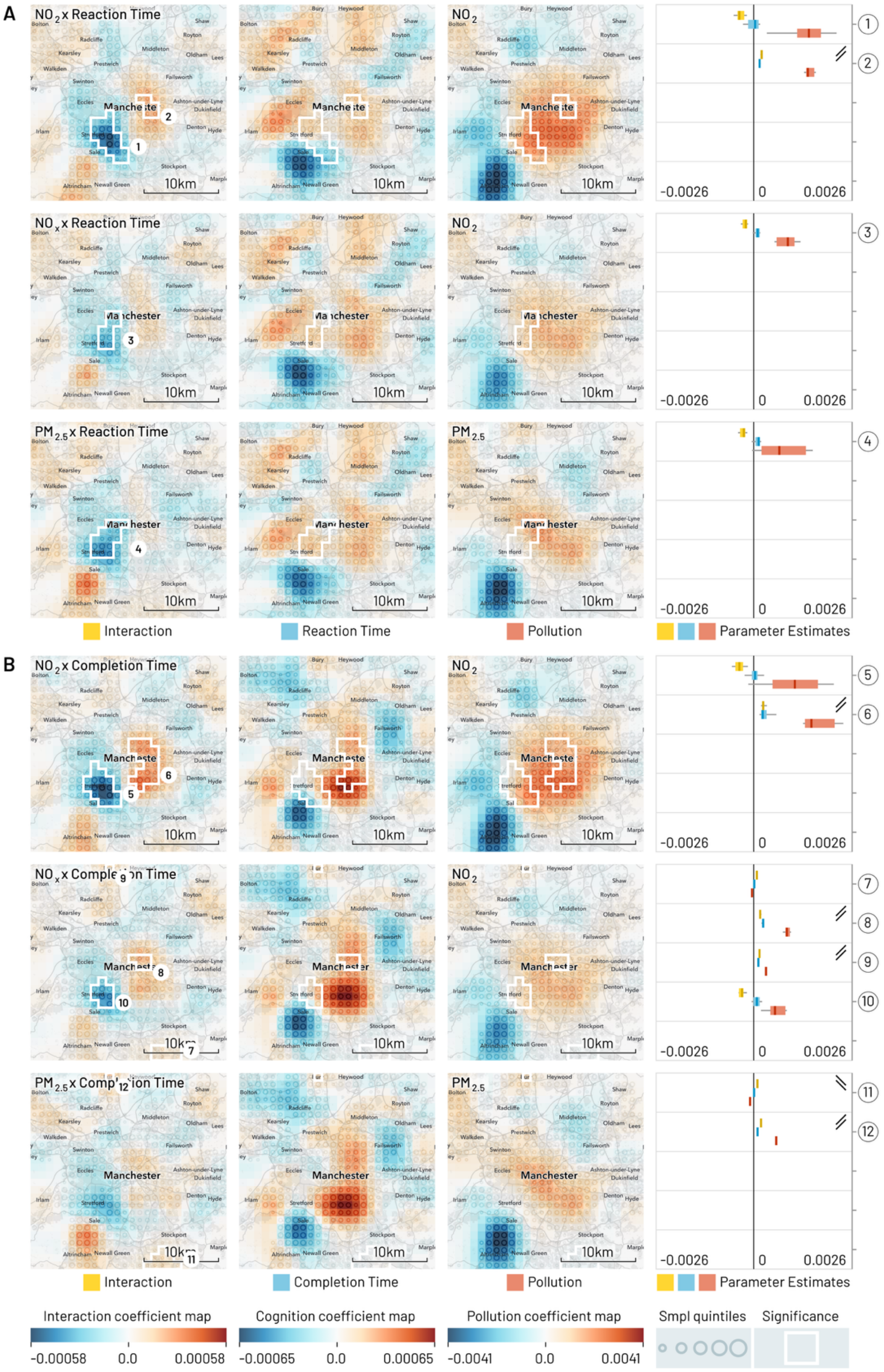

Figure S.15 Geographic regression maps showing interaction and individual effects for cognition and air pollution in Manchester with smoothing applied (95% of kernel density within a 7.5km diameter).

Sensitivity Analyses: Region Size and Position Birmingham

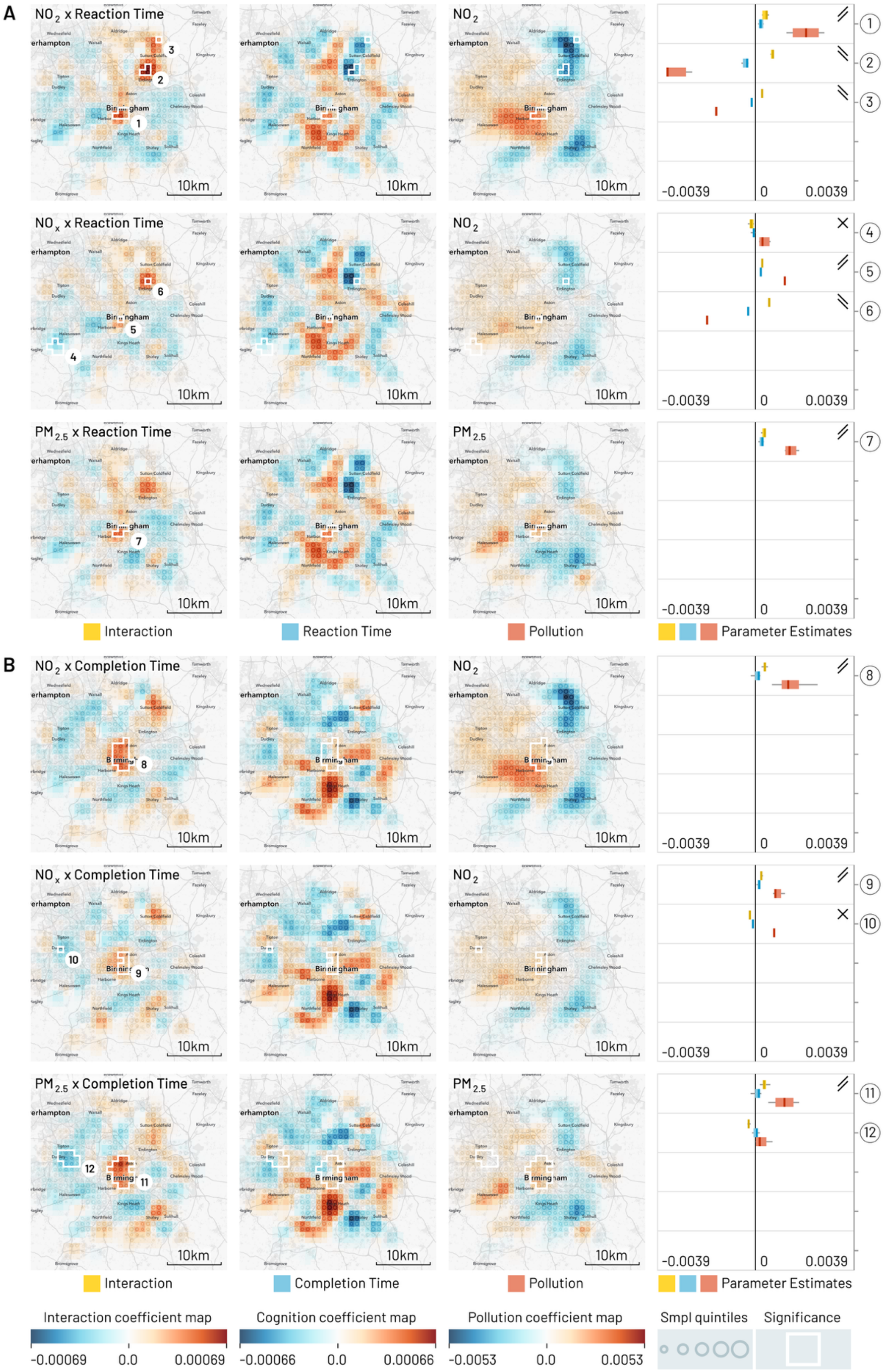

Figure S.16 Geographic regression maps showing interaction and individual effects for cognition and air pollution in Birmingham with smoothing applied (95% of kernel density within a 5km diameter). The analysis region has been expanded outward in all four cardinal directions by 5kms to capture an area 36km by 36km in size. The enlarged cohort comprises 18695 participants.

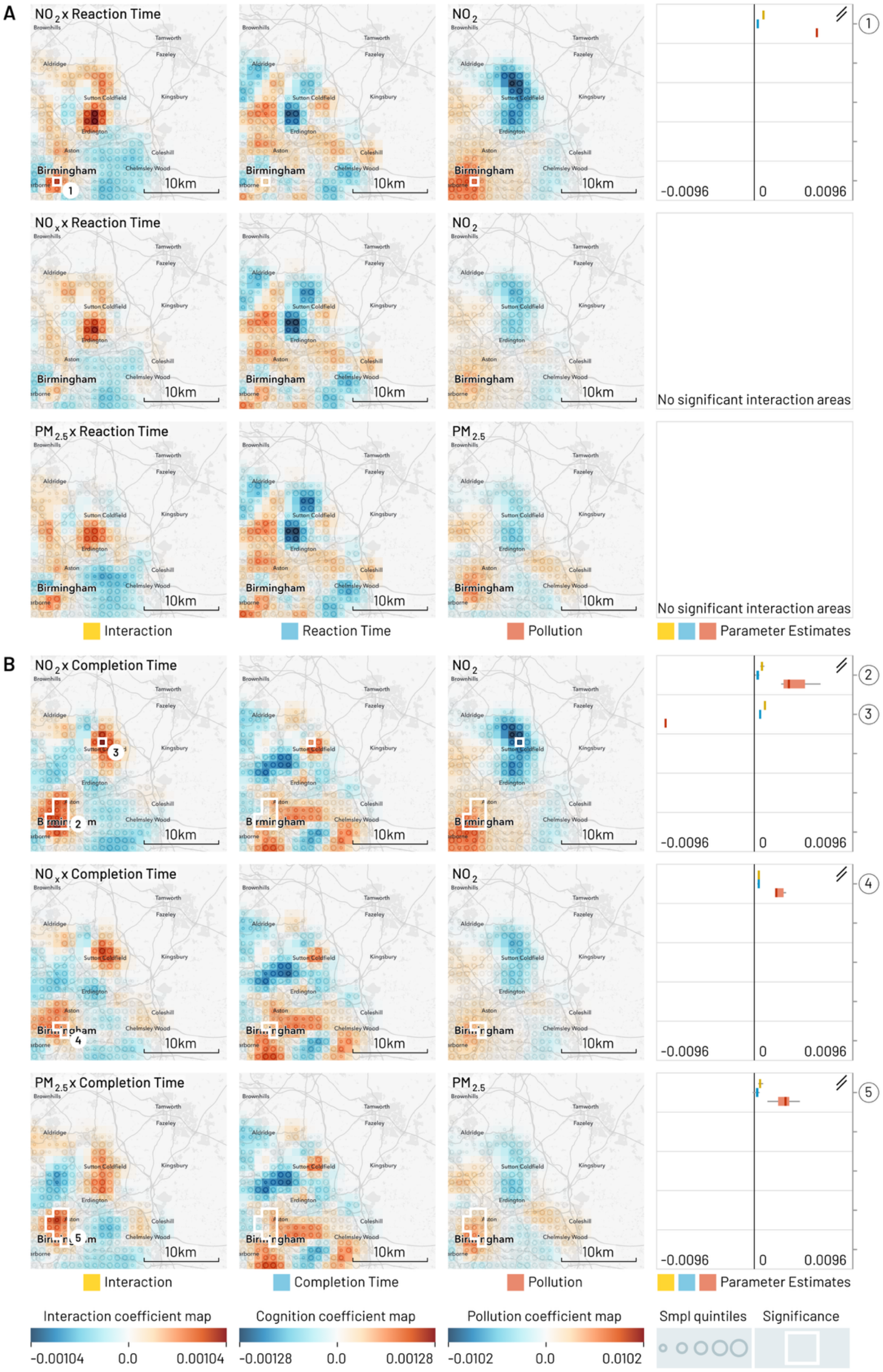

Figure S.17 Geographic regression maps showing interaction and individual effects for cognition and air pollution in Birmingham with smoothing applied (95% of kernel density within a 5km diameter). The analysis region has been shifted by 8kms along the north and east directions. The shifted cohort comprises 8746 participants.

Sensitivity Analyses: Region Size and Position Leeds

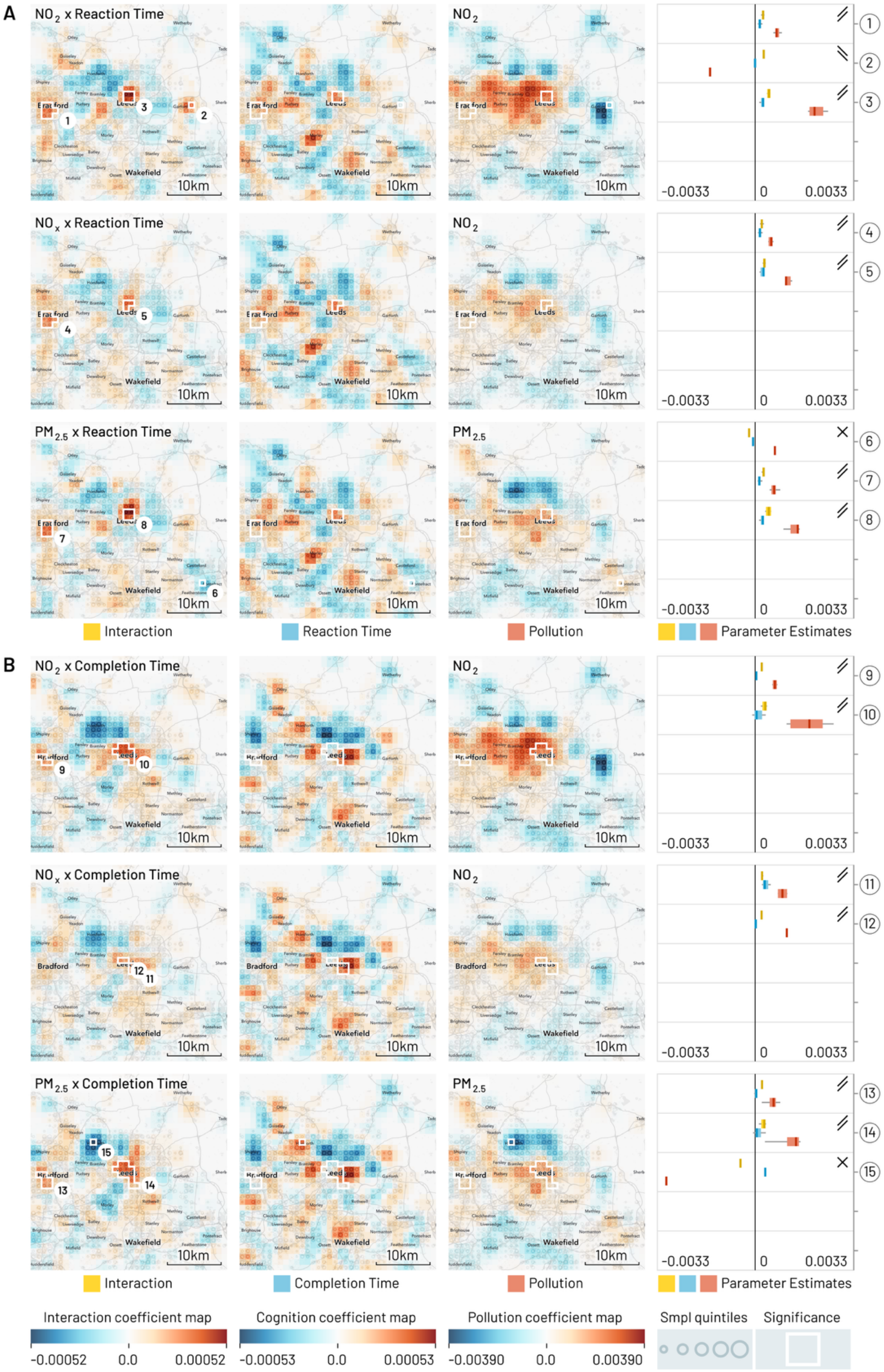

Figure S.18 Geographic regression maps showing interaction and individual effects for cognition and air pollution in Leeds with smoothing applied (95% of kernel density within a 5km diameter). The analysis region has been expanded outward in all four cardinal directions by 5kms to capture an area 36km by 36km in size. The enlarged cohort comprises 27594 participants.

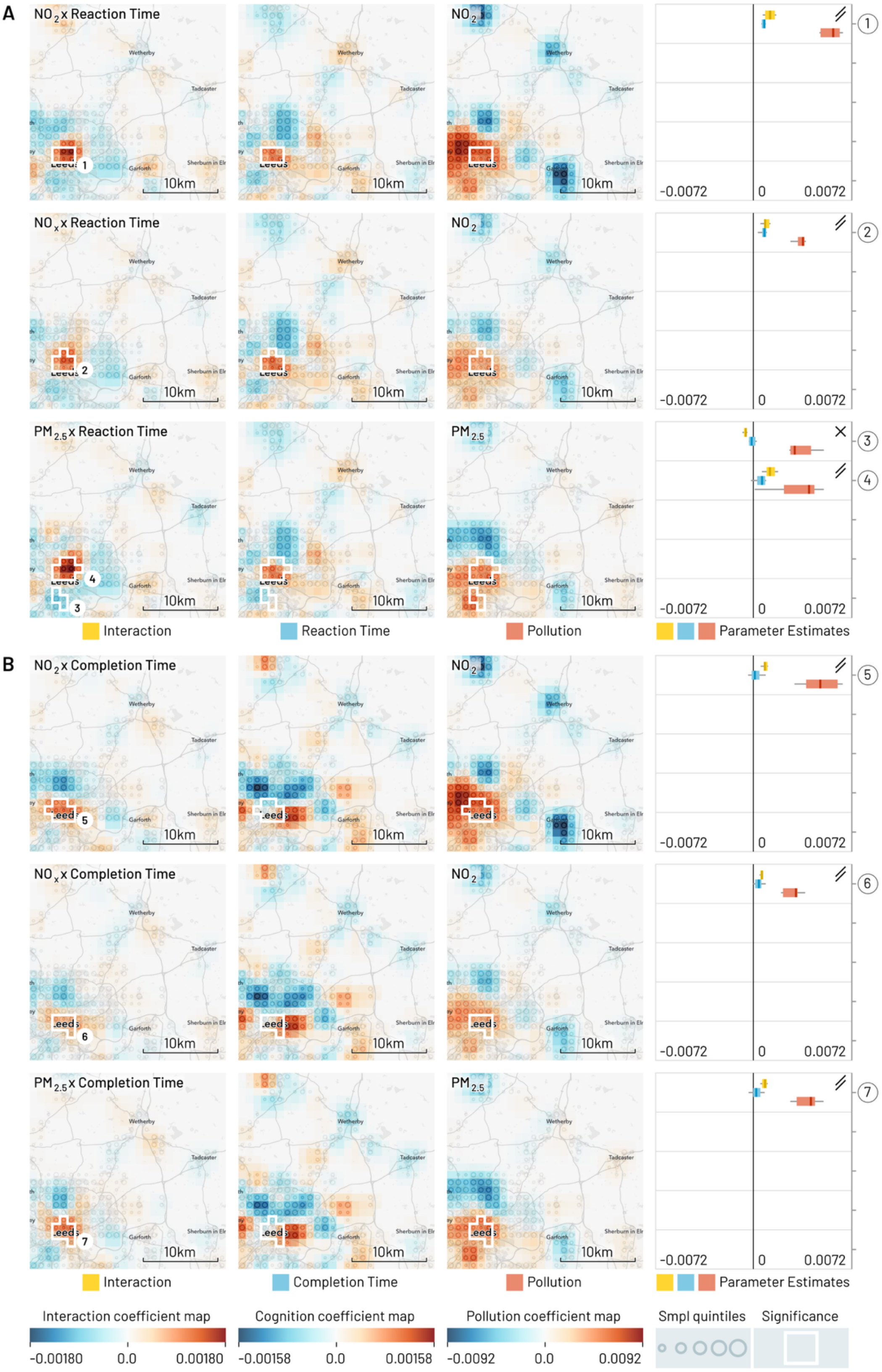

Figure S.19 Geographic regression maps showing interaction and individual effects for cognition and air pollution in Leeds with smoothing applied (95% of kernel density within a 5km diameter). The analysis region has been shifted by 8kms along the north and east directions. The shifted cohort comprises 10497 participants.

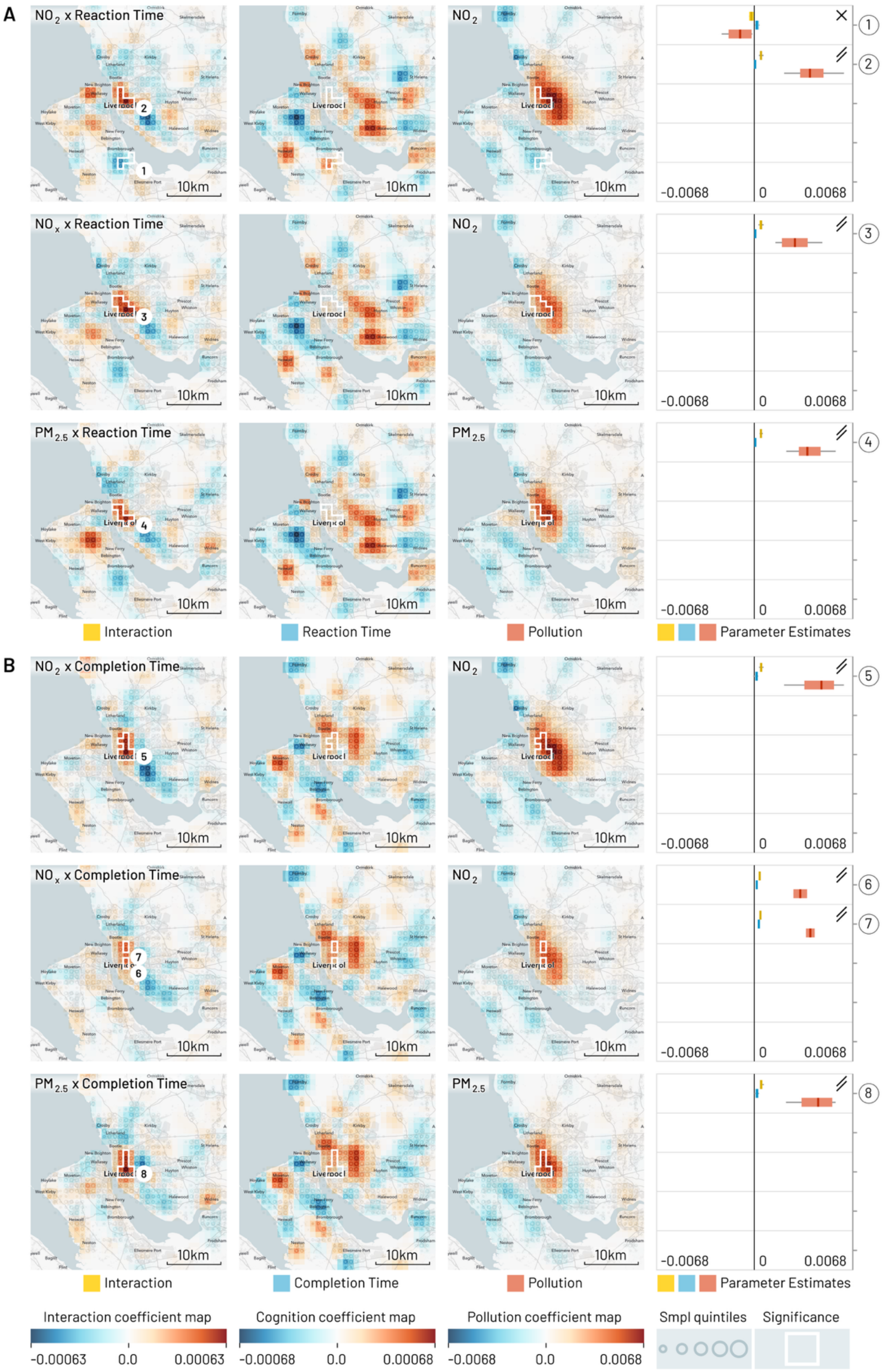

Figure S.20 Geographic regression maps showing interaction and individual effects for cognition and air pollution in Liverpool with smoothing applied (95% of kernel density within a 5km diameter). The analysis region has been expanded outward in all four cardinal directions by 5kms to capture an area 36km by 36km in size. The enlarged cohort comprises 20917 participants.

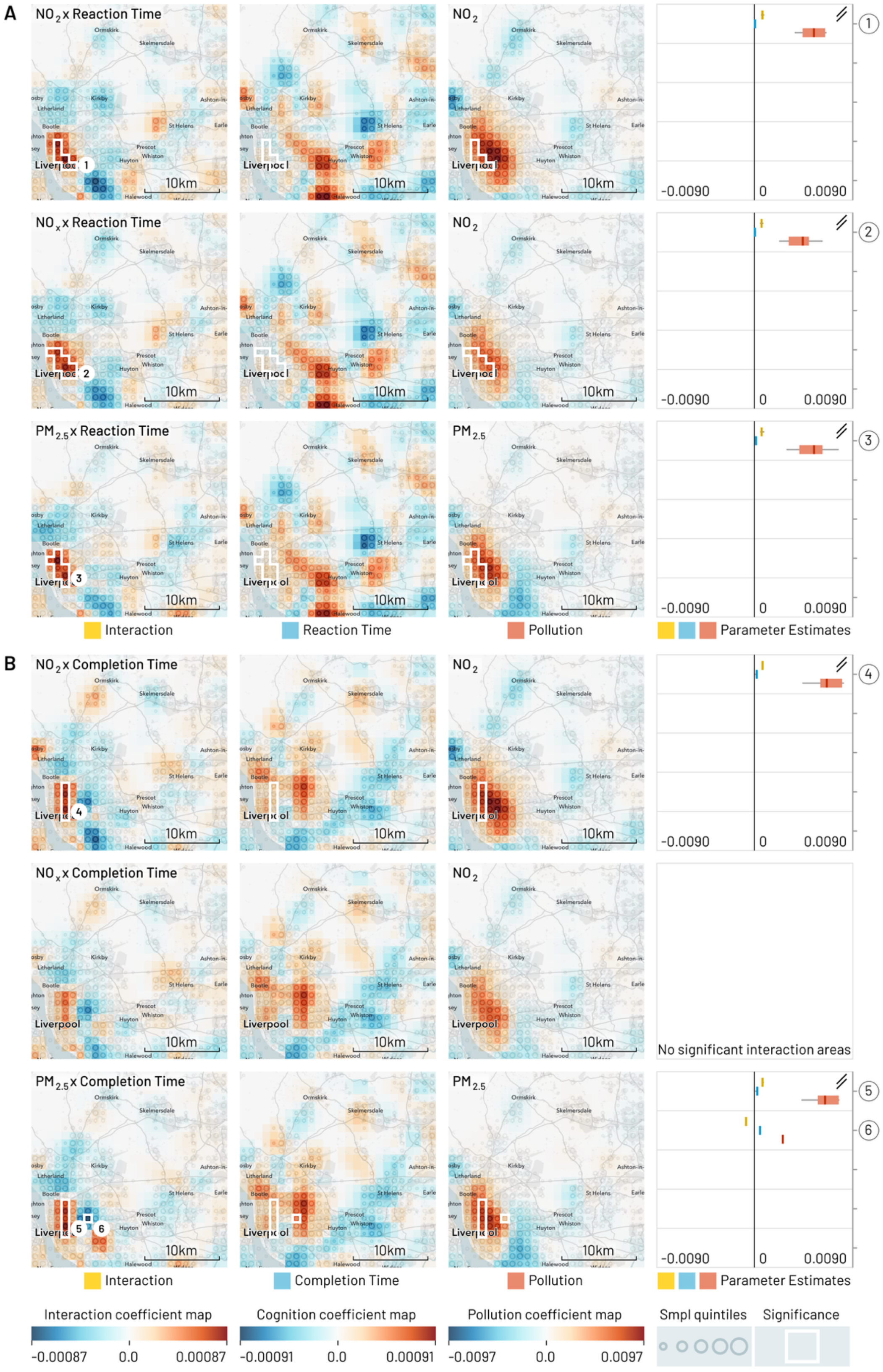

Figure S.21 Geographic regression maps showing interaction and individual effects for cognition and air pollution in Liverpool with smoothing applied (95% of kernel density within a 5km diameter). The analysis region has been shifted by 8kms along the north and east directions. The shifted cohort comprises 13531 participants.

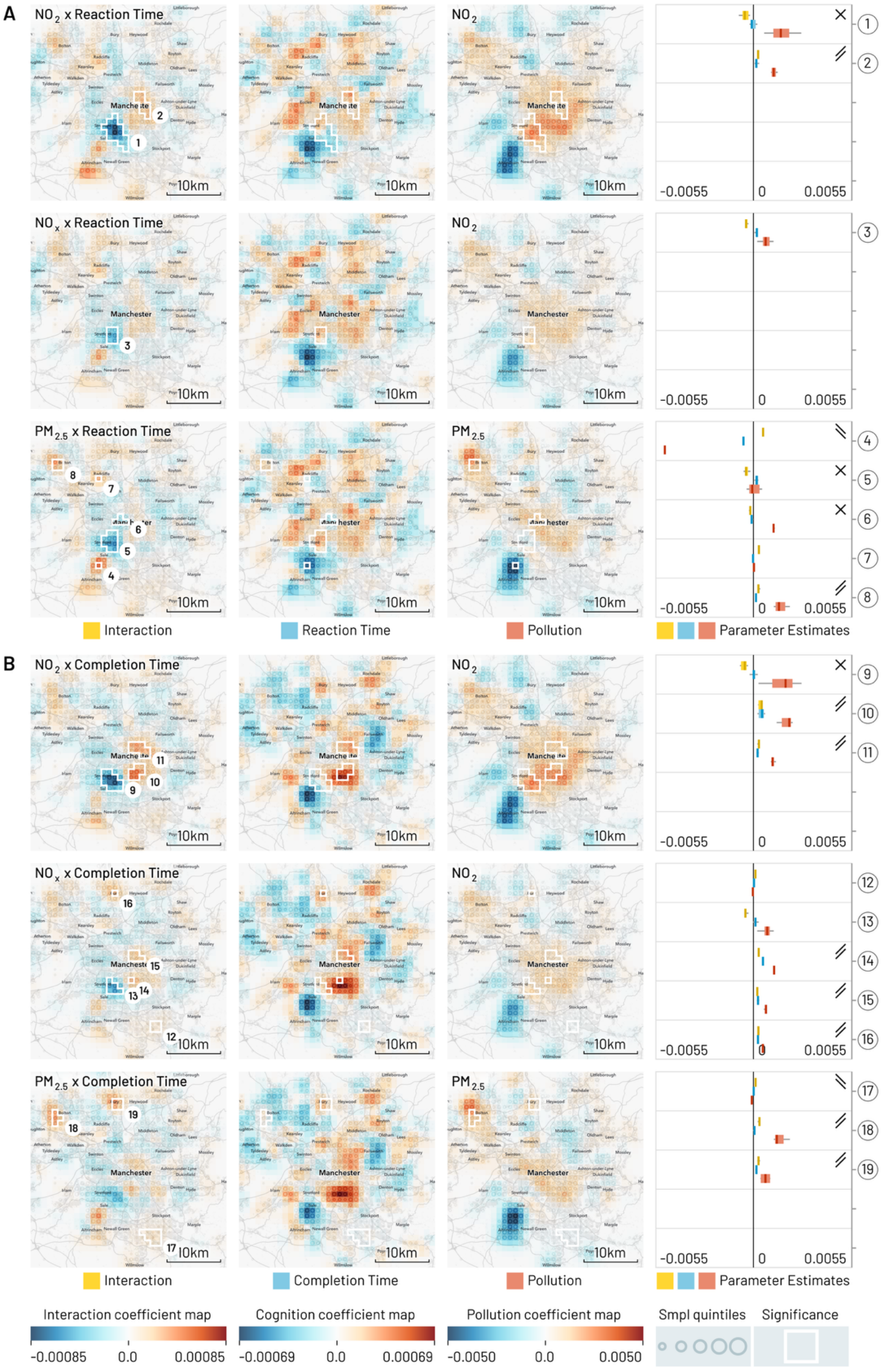

Figure S.22 Geographic regression maps showing interaction and individual effects for cognition and air pollution in Manchester with smoothing applied (95% of kernel density within a 5km diameter). The analysis region has been expanded outward in all four cardinal directions by 5kms to capture an area 36km by 36km in size. The enlarged cohort comprises 27113 participants.

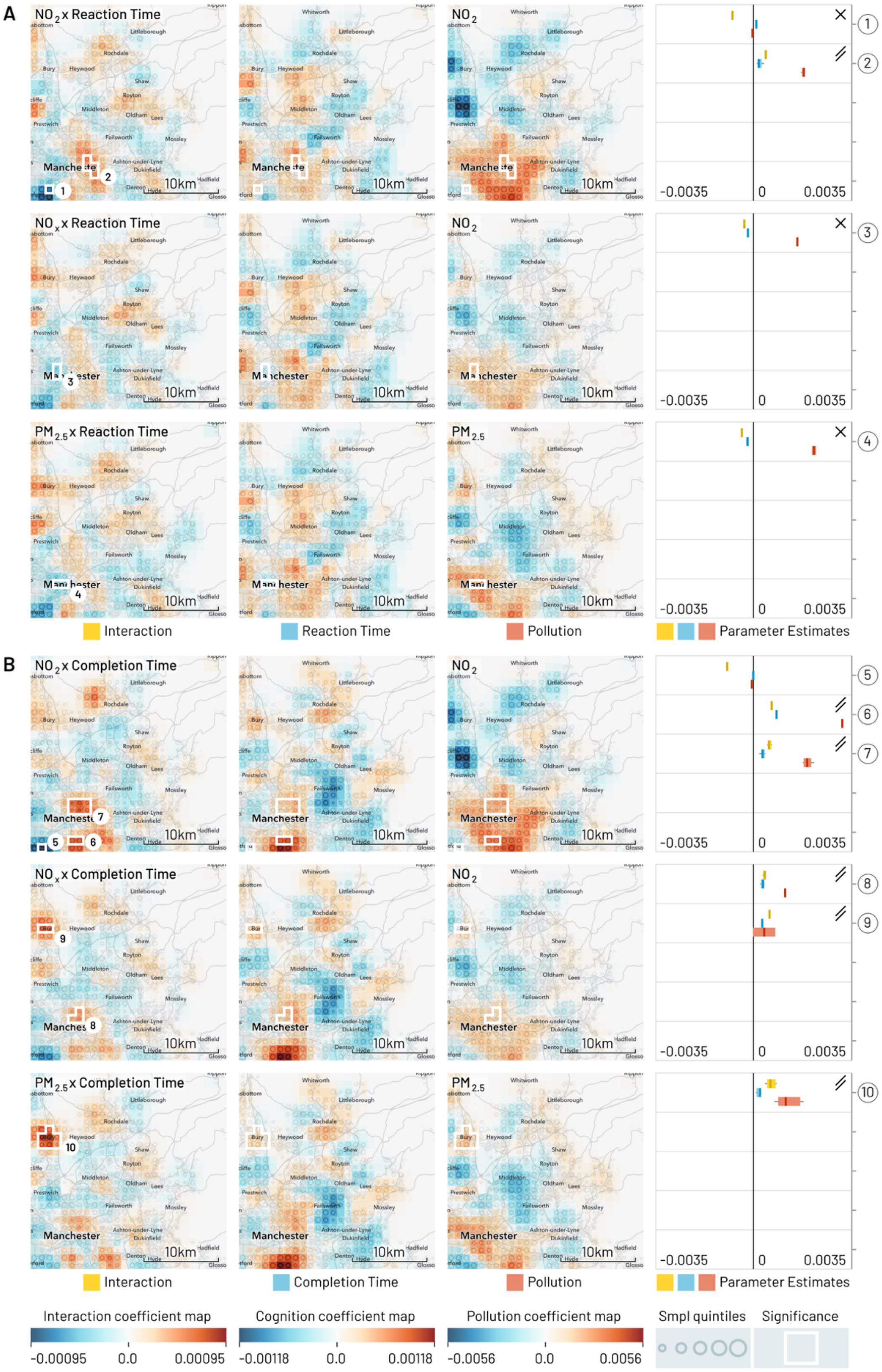

Figure S.23 Geographic regression maps showing interaction and individual effects for cognition and air pollution in Manchester with smoothing applied (95% of kernel density within a 5km diameter). The analysis region has been shifted by 8kms along the north and east directions. The shifted cohort comprises 14431 participants.

Reduced Cohort: Birmingham

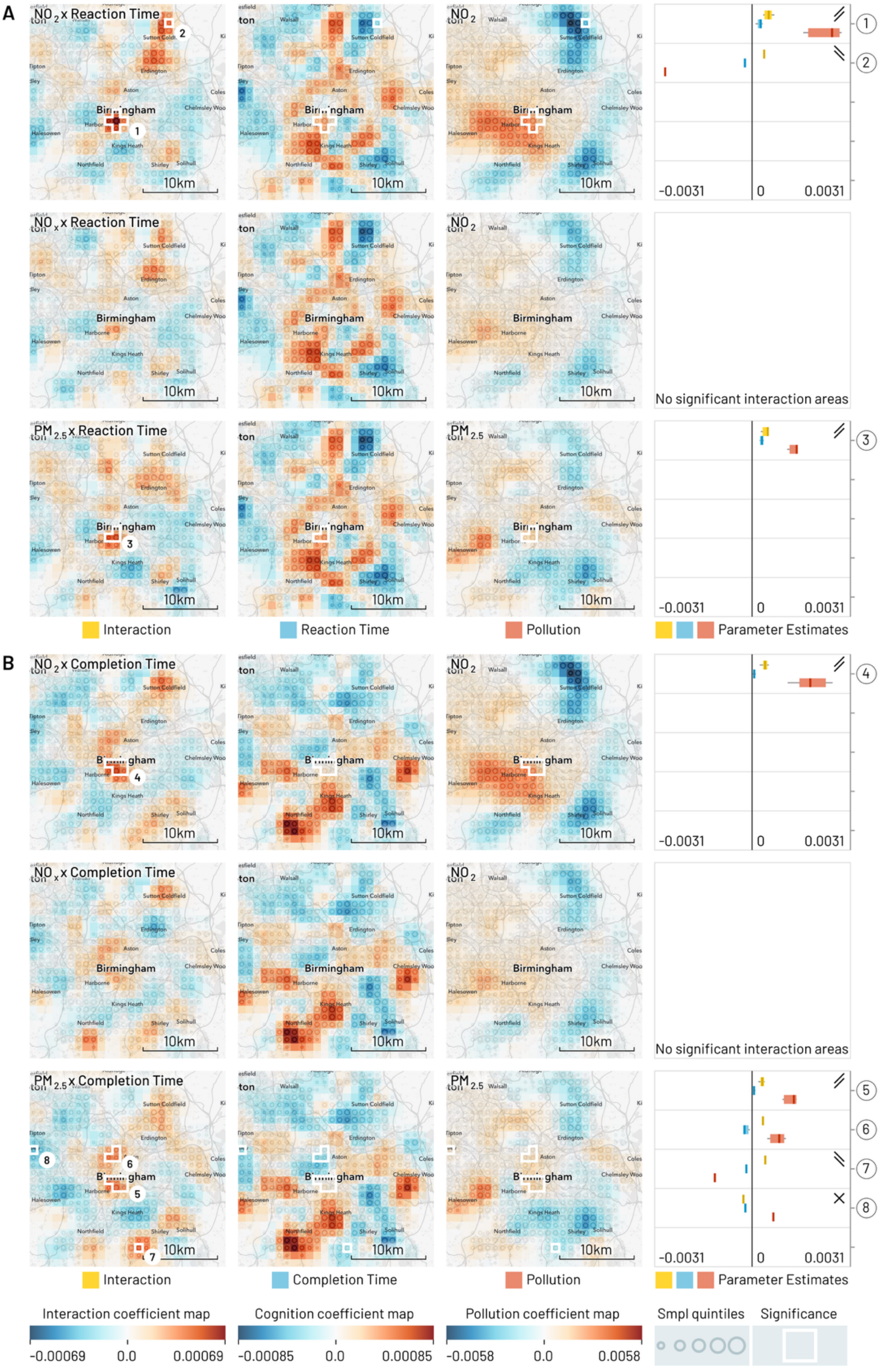

Figure S.24 Geographic regression maps showing interaction and individual effects for cognition and air pollution in Birmingham with smoothing applied (95% of kernel density within a 5km diameter). Participants with address changes have been removed from the cohort, the reduced cohort size is 13071.

Reduced Cohort: Leeds

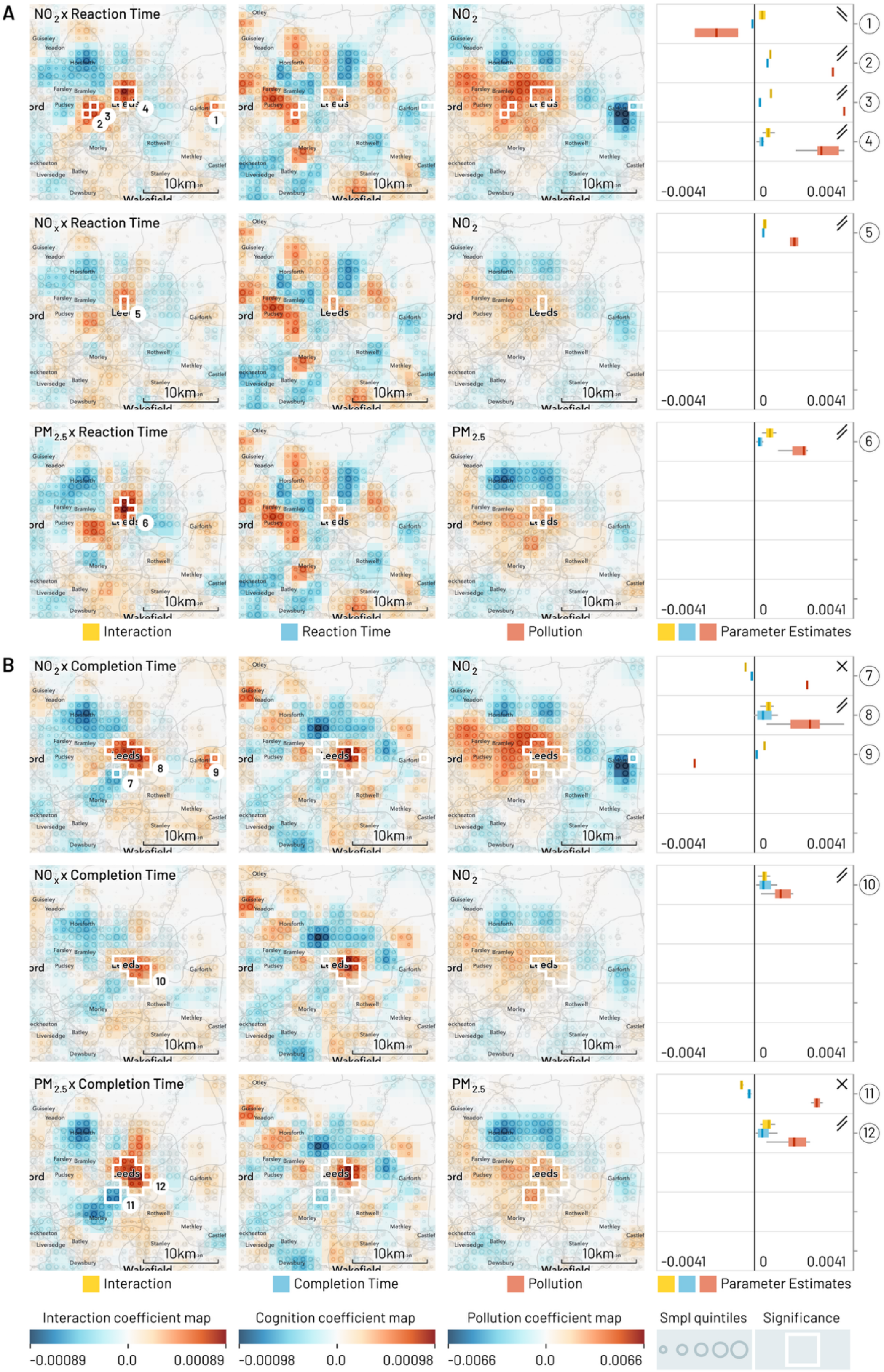

Figure S.25 Geographic regression maps showing interaction and individual effects for cognition and air pollution in Leeds with smoothing applied (95% of kernel density within a 5km diameter). Participants with address changes have been removed from the cohort, the reduced cohort size is 14389.

Reduced Cohort: Liverpool

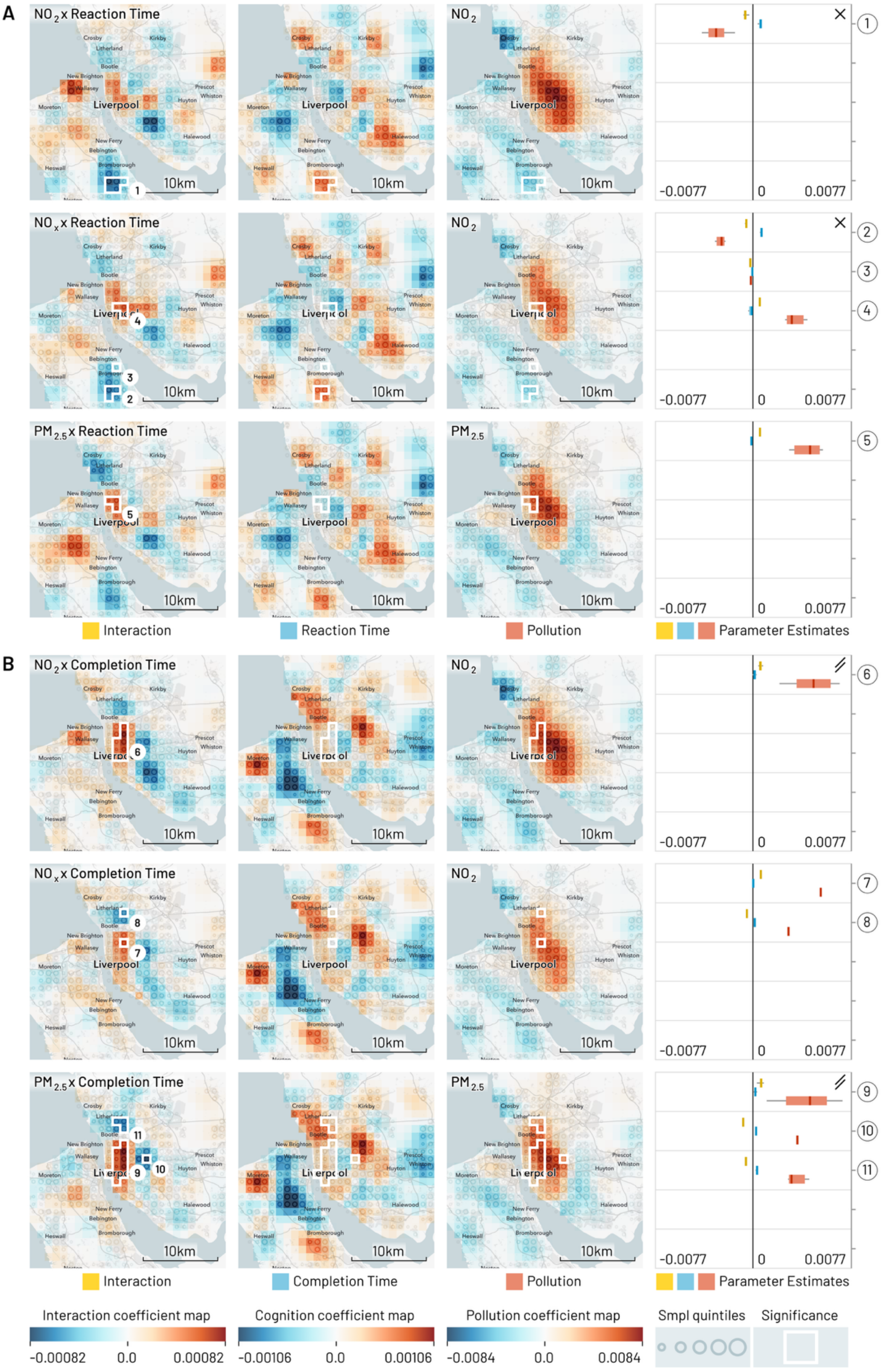

Figure S.26 Geographic regression maps showing interaction and individual effects for cognition and air pollution in Liverpool with smoothing applied (95% of kernel density within a 5km diameter). Participants with address changes have been removed from the cohort, the reduced cohort size is 12259.

Reduced Cohort: Manchester

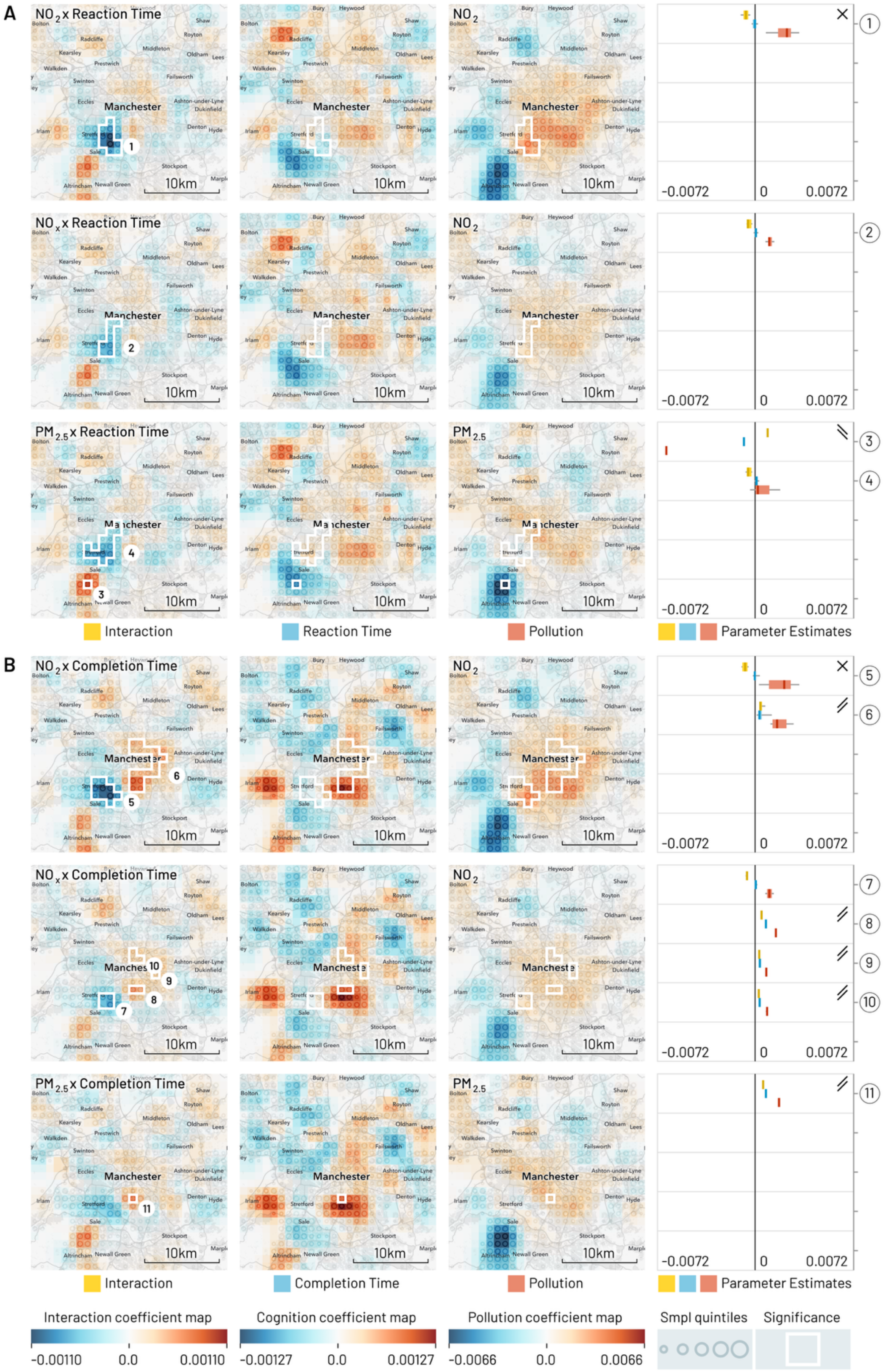

Figure S.27 Geographic regression maps showing interaction and individual effects for cognition and air pollution in Manchester with smoothing applied (95% of kernel density within a 5km diameter). Participants with address changes have been removed from the cohort, the reduced cohort size is 14735.

Geographic Context for Leeds

Figure S.28 shows the geographic context for Leeds. The grid cells that appear in most of the significant areas are in the northern part of the city centre and beyond, including Lovell Park and Little London (cf. Figure 6). The inner ring road (A64) passes through the region, and Leeds Centre monitoring station (UKA002222)[6] is located there. The average daily NO_2 concentrations for the years 2006 to 2010 computed from data provided by DEFRA's data tool [7] was 36 μg/m^3 (SD 15), while for $PM_{2.5}$ it was 14 μg/m^3 (SD 9), which are close to the limits of previous and current WHO recommendations respectively. The soon-to-be-revoked air quality management zones of Caspar House and Ebor Gardens are similarly located just north of the A64, at grid cells with 6 and 4 counts, respectively.[8] According to the latest air quality annual report for 2022, 6 stations (Bishopgate Street, Wellington Street, Joseph's Well, Neville Street, City Square and Yorkshire Post) exceeded the 40 μgm/m^3 UK air quality objective.[9] Although no precise location for these stations is provided in the report, a corresponding search of road and place names indicates that these are all central locations north of the river (except for the Yorkshire Post) and all of them coincide with grid cells that are significant in 2 or 3 analyses.

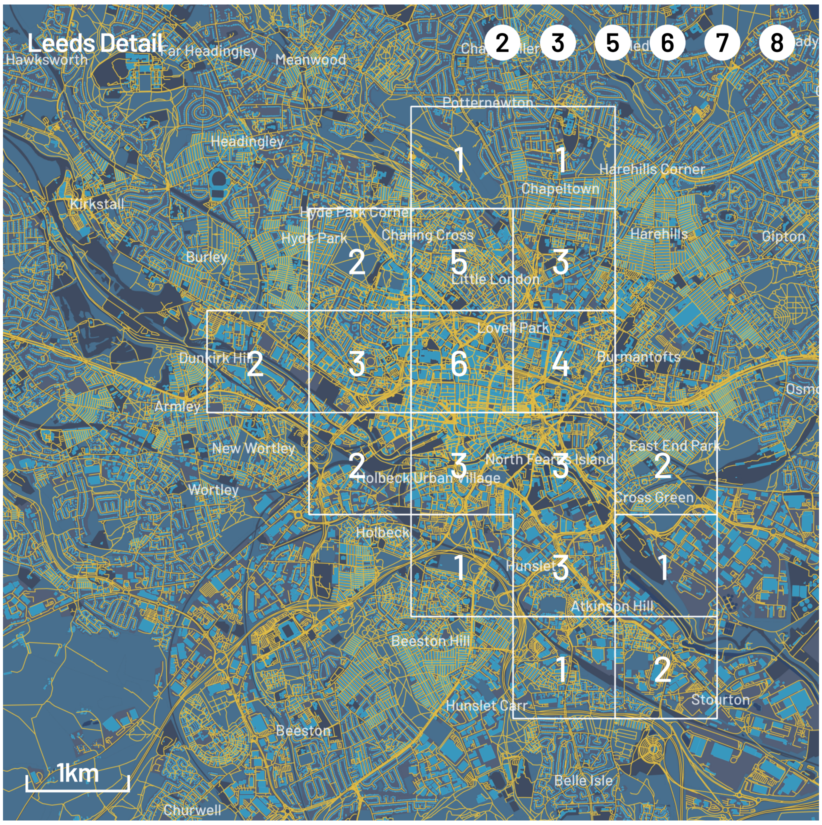

Figure S.28. Detail of geographic context for Leeds for a subset of the significant areas shown in Figure 2. Their identifying numbers are listed in the top right and their outlines delineated in white. All regions exhibit a positive interaction and individual effects that indicate the co-occurrence of reduced cognition and increased air pollution. Each grid cell indicates the count of significant areas it is part of. Grid cells with higher counts contain primary roads and are located in central areas. Geographic data provided by OpenStreetMap.

Geographic Context for Liverpool

The entire City of Liverpool has been declared an air quality management area in 2009.[10] Liverpool city council was also directed in 2018 by the UK government to develop a feasibility study to reduce roadside NO_2 concentrations.[11] The pollution climate mapping model (PCM) developed by the Department for Environment Food & Rural Affairs projected six road links in exceedance of the legal limit, five of which in central locations.[12] These road links are identified in the feasibility study submitted by the council.[13] Four of these five links are within areas also identified by our analysis, in the centre of Liverpool, in St. George's Quarter and New Islington along the A580, A57 (Dale Street) and A5038 (Lime Street). In addition, our analysis includes other primary routes, such as the A57 (Scotland Road and Kingsway), more of the A580 (Erskine Street and Low Hill), as well as the A5047 (Edge Lane). The Kirkdale South and Vauxhall census ward bordering the A57 shows that only 32.7% of households are not deprived in any dimension,[14] hinting at the socio-economic dimension of pollution and its effects.

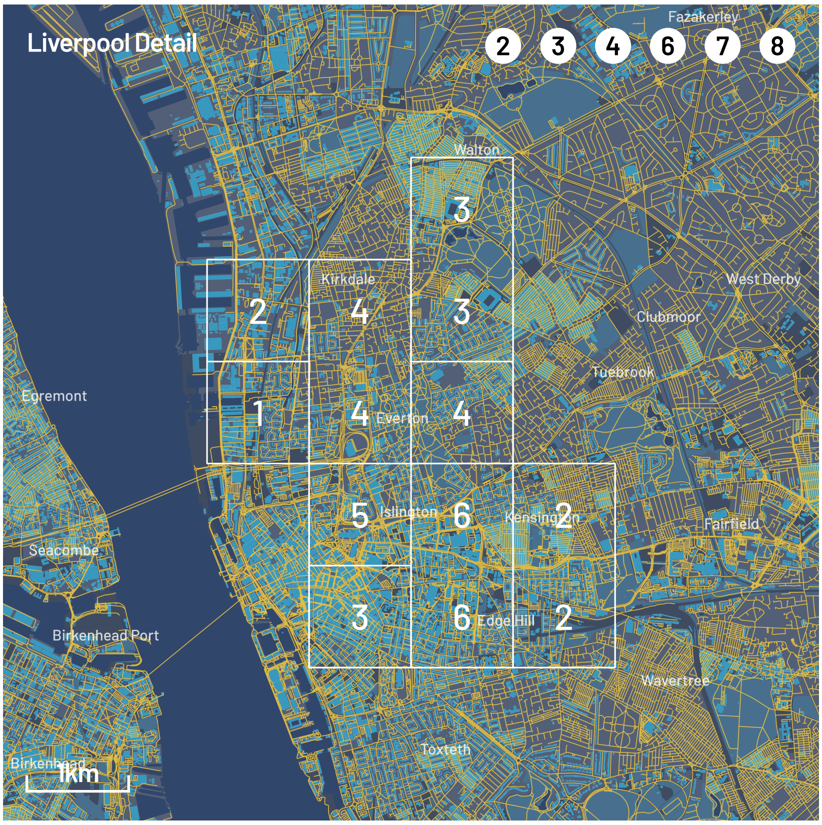

Figure S.29. Detail of geographic context for Liverpool for a subset of the significant areas shown in Figure 3. Their identifying numbers are listed in the top right and their outlines delineated in white. All regions exhibit a positive interaction and individual effects that indicate the co-occurrence of reduced cognition and increased air pollution. Each grid cell indicates the count of significant areas it is part of. Grid cells with higher counts contain primary roads and are located in central areas. Geographic data provided by OpenStreetMap.

Geographic Context for Manchester

In Manchester, we find that significant grid cells are in areas northeast of the centre, adjacent to the A34/A665 ring road (Figure S.30A) covering the neighbourhoods of Strangeways, Collyhurst, Ancoats and New Islington. In addition, two zones detached from the centre can be distinguished: in the east, covering the areas of Belle Vue and Longsight, and in the south, around Moss Side and Rusholme. The former is bisected by the A6010 Pottery Lane ring road, the latter contains the A5103 Princess Road and the A6010 Wilbraham Road primary roads. When comparing the locations of Manchester's NO_2 monitoring stations with these zones,[15] one finds that a majority of them had annual mean measurements in the top quartile of all stations in Manchester within the first zone northeast of the centre. There is only one such site in the second zone, whereas the third zone contains no measurement sites. In 2023, the local authorities of Manchester published a clean air plan to reduce NO_2 emissions on the A58, Bolton Road, Bury, a market town northwest of Manchester in response to a government request.[16] Interestingly, Bury is also one of the areas identified by our analysis for completion time and nitrogen oxides and particulate matter (Figure S.30B).

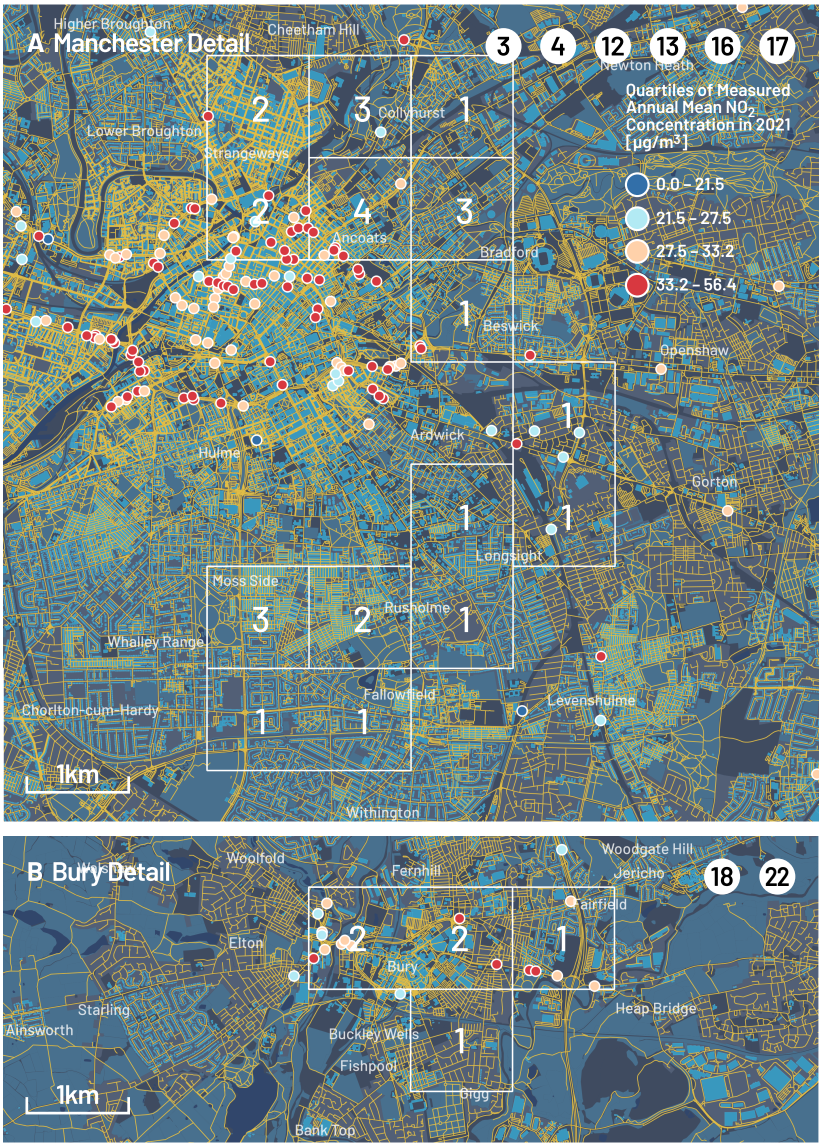

Figure S.30. Detail of geographic context for Manchester for a subset of the significant areas shown in Figure 4. Their identifying numbers are listed in the top right and their outlines delineated in white. All regions exhibit a positive interaction and individual effects that indicate the co-occurrence of reduced cognition and increased air pollution. Each grid cell indicates the count of significant areas it is part of. The monitoring sites for the Manchester clean air plan and local air quality management are marked as dots and colour-coded by the measured annual mean NO_2 concentration quartiles for 2021. Grid cells with higher counts contain primary roads and are located in central areas. There is also a noticeable overlap with monitoring sites in the top quartile. Geographic data provided by OpenStreetMap. Panel (A) Central Manchester, panel (B) Bury market town.

References for Geographic Context

1 Department for Environment Food & Rural Affairs. Air Quality Plan for tackling roadside nitrogen dioxide concentrations in West Midlands Urban Area (UK0002). 2017; published online July. https://uk-air.defra.gov.uk/assets/documents/no2ten/2017-zone-plans/AQplans_UK0002.pdf (accessed Jan 21, 2025).

2 Birmingham City Council. 2019 Air Quality Annual Status Report (ASR). 2019; published online Nov 30. https://www.birmingham.gov.uk/downloads/file/15061/air_quality_annual_status_report_2019_containing_data_for_2018.

3 Birmingham City Council. Birmingham City Council Air Quality Action Plan. 2021; published online Feb. https://www.birmingham.gov.uk/downloads/download/4061/birmingham_city_council_air_quality_action_plan_2021-2026 (accessed Jan 21, 2025).

4 Birmingham City Council. Payments for the Clean Air Zone go live. https://www.birmingham.gov.uk/news/article/888/payments_for_the_clean_air_zone_go_live (accessed Jan 21, 2025).

5 Liu B, Bryson JR, Sevinc D, *et al.* Assessing the Impacts of Birmingham's Clean Air Zone on Air Quality: Estimates from a Machine Learning and Synthetic Control Approach. *Environ Resource Econ* 2023; **86**: 203–31.

6 Department for Environment Food & Rural Affairs. Site Information for Leeds Centre (UKA00222). https://uk-air.defra.gov.uk/networks/site-info?site_id=LEED (accessed Jan 21, 2025).

7 Department for Environment Food & Rural Affairs. Data Selector. https://uk-air.defra.gov.uk/data/data_selector_service (accessed Jan 21, 2025).

8 Leeds City Council. Leeds' air quality continues to improve, new data shows. Leeds City Council News. 2023; published online June 15. https://news.leeds.gov.uk/news/leeds-air-quality-continues-to-improve-new-data-shows (accessed Jan 21, 2025).

9 Leeds City Council. Air quality annual status report executive summary. 2022. https://www.leeds.gov.uk:443/clean-air/air-quality-annual-report (accessed Jan 21, 2025).

10 Department for Environment Food & Rural Affairs. Liverpool City AQMA. 2009; published online April 1. https://uk-air.defra.gov.uk/aqma/details?aqma_ref=211 (accessed Jan 21, 2025).

11 Department for Environment Food & Rural Affairs. Supplement to the UK plan for tackling roadside nitrogen dioxide concentrations: local authorities feasibility studies. https://uk-air.defra.gov.uk/library/no2ten/2018-la-tfs-documents (accessed Jan 21, 2025).

12 Department for Environment Food & Rural Affairs. Air modelling for Defra. https://uk-air.defra.gov.uk/research/air-quality-modelling?view=modelling (accessed Jan 21, 2025).

13 Liverpool City Council. Targeted feasibility study to deliver nitrogen dioxide concentration compliance in the shortest possible time. 2018; published online Oct. https://uk-air.defra.gov.uk/library/assets/documents/no2ten/Liverpool_FINAL.pdf (accessed Jan 21, 2025).

14 Office for National Statistics. Kirkdale South & Vauxhall: Household deprivation. 2021; published online March. https://www.ons.gov.uk/census/maps/choropleth/population/household-deprivation/hh-deprivation/household-is-not-deprived-in-any-dimension (accessed Jan 21, 2025).

15 Greater Manchester Combined Authority (GMCA). Greater Manchester LAQM and CAP diffusion tube results 2011-2021. https://assets.ctfassets.net/tlpgbvy1k6h2/5hlsNqrLHKdPYissl03llt/faba6f8e46c99796fb460d6e3d11b619/GM_LAQM_and_CAP_diffusion_tube_results_2011-2021.xlsx (accessed Jan 21, 2025).

16 Transport for Greater Manchester. Approach to Address Persistent Exceedances Identified on the A58 Bolton Road, Bury. 2023; published online March. https://democracy.greatermanchester-ca.gov.uk/documents/s24939/Appendix%203.%20GM%20CAP%20A58%20Bury%20Measure%20Report%20DRAFT%20for%20AQAC%20Approval%20Feb%2023.pdf.
